# Inflammatory mechanisms underlying early Alzheimer’s disease pathology: evidence from the aging rhesus macaque brain

**DOI:** 10.3389/fncel.2026.1750092

**Published:** 2026-02-12

**Authors:** Dibyadeep Datta, Min Wang, Amy F. T. Arnsten

**Affiliations:** 1 Department of Psychiatry, Yale Medical School, New Haven, CT, United States; 2 Department of Neuroscience, Yale Medical School, New Haven, CT, United States

**Keywords:** Alzheimer’s disease, calcium, cognition, GCPII, glutamate, inflammation, primate, pyramidal cell

## Abstract

Inflammation plays a large role in the etiology of the late onset, sporadic form of Alzheimer’s disease (AD), yet these critical factors are not adequately modeled in mice where inflammatory mechanisms often differ widely from primates. In contrast, aging rhesus macaques offer a powerful translational model for investigating how advancing age and inflammation initiate early-stage pathology in sporadic AD, and for evaluating preventive therapeutic strategies. Unlike rodents, macaques possess highly developed association cortices with magnified calcium signaling, human-like inflammatory responses, and are naturally homozygous for ApoE-ε4—factors that together contribute to the spontaneous emergence of tau and amyloid pathology alongside cognitive decline. Critically, macaques allow the detection of early, soluble forms of hyperphosphorylated tau (pTau), including pT217Tau, which rapidly dephosphorylates postmortem and is rarely observable in human brain tissue outside of biopsies. New findings reveal that soluble pTau is neurotoxic and capable of propagating pathology across cortical networks, with elevated pT217Tau in plasma. Growing evidence points to age-related inflammatory signaling as a key driver of calcium dysregulation, which in turn promotes tau hyperphosphorylation, amyloid-β (Aβ) accumulation, synapse loss and autophagic degeneration. Both GCPII (glutamate carboxypeptidase II) and kynurenic acid inflammatory signaling have expanded roles in the primate association cortices that contribute to cognitive deficits. Pharmacological interventions in aged macaques demonstrate that targeting inflammation and restoring calcium homeostasis can significantly reduce pTau pathology with minimal side effects—highlighting a promising path for early intervention in AD.

## Introduction

Sporadic Alzheimer’s disease (sAD) unfolds over decades as genetic and environmental factors amplify neuroinflammatory signaling, accelerating pathology in the aging brain. Preventive treatments therefore hinge on pinpointing the earliest inflammation-driven triggers of disease. Traditional mouse models fall short because rodent immune responses and cortical architecture diverge markedly from those of humans (King, 2018). Aging rhesus macaques, by contrast, share human-like association cortices and immune pathways and spontaneously develop tau and amyloid pathology, synaptic loss, and cognitive decline, including age-related activation of critical glial cell-types such as microglia and astrocytes (Arnsten et al., 2021; Paspalas et al., 2018; Beckman et al., 2021; Beckman et al., 2019). Crucially, they allow direct study of the initial, soluble phase of hyperphosphorylated tau (pTau) — including pT217Tau — that is neurotoxic and capable of propagating pathology across cortical networks (Barthelemy et al., 2020; Palmqvist et al., 2020; Datta et al., 2024). These early-stage, soluble pTau species degrade almost immediately post-mortem in humans (Matsuo et al., 1994; Wang et al., 2015), limiting research to rare biopsy samples, whereas they can be sampled *ex vivo* from macaque brain and plasma and visualized with high-resolution imaging (Datta et al., 2024). Recent macaque studies reveal that age-related inflammatory cascades disrupt calcium homeostasis, drive tau hyperphosphorylation, and spur Aβ production; conversely, anti-inflammatory and calcium-modulating therapies markedly lessen pathology, pointing to viable strategies for early intervention (Datta and Arnsten, 2025; Arnsten et al., 2019; Arnsten et al., 2021; Datta and Arnsten, 2025). Because macaques are naturally homozygous for ApoE-ε4, insights from this model are especially pertinent for developing treatments tailored to ApoE-ε4 carriers, who have responded poorly to current Aβ- and tau-directed antibodies (van Dyck et al., 2023; Congdon et al., 2023), underscoring the need for alternative, upstream therapeutic strategies in appropriate model systems.

## Tau and amyloid pathology in AD

AD is defined by two key neuropathological features: extracellular amyloid-β (Aβ) plaques and intracellular neurofibrillary tangles (NFTs) composed of hyperphosphorylated tau. These processes are interconnected—Aβ oligomers can promote tau phosphorylation (Um et al., 2013), while phosphorylated tau aggregates may, in turn, enhance Aβ production, creating a self-reinforcing pathological loop (Arnsten et al., 2021; Paspalas et al., 2018). Notably, cognitive decline in AD correlates more strongly with the presence of NFTs than with Aβ plaques (Nelson et al., 2012), highlighting the importance of understanding how tau phosphorylation develops in aging association cortices.

Tau, a microtubule-associated protein encoded by *MAPT*, is generated in six splice variants that differ by the presence of 0, 1, or 2 N-terminal inserts (0 N, 1 N, 2 N) and by having either three or four microtubule-binding repeats (3R or 4R) at the C-terminal end. Structurally, tau contains an N-terminal projection domain, a proline-rich segment, the microtubule-binding region (with its 3 or 4 tandem repeats), and a short C-terminal tail (Fitzpatrick et al., 2017; Mandelkow and Mandelkow, 1998). Under normal conditions, tau stabilizes microtubules, but under pathological conditions, tau protein undergoes an extensive array of post-translational modifications: numerous kinases heavily phosphorylate residues in the proline-rich region; additional regulation comes from acetylation, methylation, ubiquitination, and proteolytic truncation. Collectively, these modifications disrupt tau’s normal microtubule-stabilizing functions and heighten its propensity to aggregate in neurodegenerative diseases (Fitzpatrick et al., 2017; Mandelkow and Mandelkow, 1998; Rauch et al., 2020; Rissman et al., 2004), ultimately forming fibrils within dendrites that eventually accumulate in the soma as NFTs. This pathological process is accompanied by neuronal death via autophagic degeneration, leaving behind characteristic “ghost tangles” (Arnsten et al., 2021).

In individuals with sAD, cortical tau pathology initially emerges in layer II of the transentorhinal and entorhinal cortices (ERC), corresponding to Braak Stages I–II (Hyman et al., 1984; Braak et al., 2011). From there, it spreads to interconnected limbic and association cortical areas, as well as the hippocampus, during Braak Stages III–IV. The layer II cell islands of the ERC serve as a critical hub, channeling input from widespread association cortices into the hippocampus to support new memory formation (Hyman et al., 1984). This region’s anatomical importance makes it especially vulnerable as a site where tau pathology begins to seed and propagate through cognitive and memory circuits (Kaufman et al., 2018). As the disease progresses, tau aggregates are found in regions such as the dorsolateral prefrontal cortex (dlPFC), which is essential for abstract reasoning, working memory, and executive functions—changes that correlate with cognitive impairment (Giannakopoulos et al., 2003). By Braak Stage V, tau pathology becomes widespread in association cortices but does not affect primary sensory areas, such as visual and auditory cortices, until the final stage of the disease (Braak Stage VI) (Braak et al., 2011; Lewis et al., 1987). This spatial progression of pathology mirrors the clinical course of AD, beginning with recent memory loss and expanding to broader cognitive decline and long-term memory impairment, while primary sensory-motor functions are largely preserved until late stages.

Amyloid beta (Aβ) peptides are generated through the sequential cleavage of amyloid precursor protein (APP) by β-secretase followed by *γ*-secretase (LaFerla et al., 2007). This process is accelerated within endosomes that contain β-secretase (Grynspan et al., 1997), and may be further intensified by the presence of the ApoE-ε4 genotype. In contrast, when APP is localized to the plasma membrane, it is more commonly processed by *α*-secretase, which leads to its degradation rather than Aβ production (LaFerla et al., 2007). Because Aβ is primarily released into the extracellular space, it is likely more readily detected in cerebrospinal fluid (CSF) and plasma compared to phosphorylated tau (pTau). As extracellular Aβ accumulates, monomers begin to aggregate into oligomers, then protofibrils, and eventually form insoluble fibrillar plaques. Amyloid pathology first emerges in the association areas of the temporal neocortex and later spreads to other regions of the neocortex (Braak and Braak, 1991). As AD advances, Aβ plaques also accumulate in subcortical structures such as the striatum and thalamus [reviewed in Braak and Del Trecidi (2015)]. This distribution pattern aligns with the idea that Aβ is released from the axons of neurons affected by pTau, with higher-order association cortices potentially contributing Aβ to their downstream projection targets (Braak and Del Trecidi, 2015; Braak and Del Tredici, 2015).

## Aging rhesus macaques exhibit the same pattern and sequence of sAD pathology

Comprehensive anatomical studies have shown that aging rhesus macaques exhibit a qualitatively similar pattern and progression of tau pathology to that seen in humans, across subcellular, cellular, and regional levels (Arnsten et al., 2019; Arnsten et al., 2021). For instance, the spatial and temporal development of tau pathology in rhesus macaque’s echoes that of sAD in humans—beginning in the layer II cell islands of the ERC, later appearing in the dlPFC, but notably sparing V1 cortices. In both species, tau phosphorylation originates in the distal dendrites and dendritic spines and gradually advances into the neuronal soma (Paspalas et al., 2018; Datta and Arnsten, 2025; Braak and Del Trecidi, 2015; Braak and Braak, 1991; Braak and Del Tredici, 2011). This pattern is especially evident with early, soluble phosphorylated tau epitopes such as pS214Tau and pT217Tau in rhesus macaques at the nanoscale-level with high spatial resolution ultrastructural studies (Paspalas et al., 2018; Datta et al., 2024; Carlyle et al., 2014). In fact, elevated plasma levels of pT217Tau are now considered a promising early biomarker that predicts future AD (Palmqvist et al., 2020; Barthélemy et al., 2024; Mendes et al., 2024; Pandey et al., 2025), and the macaque model provides key insights not possible in other species. Research in aging rhesus monkeys has revealed that early-stage, soluble pS214Tau and pT217Tau can traffic between neurons, particularly near or within glutamatergic synapses, potentially enabling the spread of tau pathology across networks of higher-order cortical glutamatergic neurons (Paspalas et al., 2018; Datta et al., 2024; Carlyle et al., 2014). This synaptic tau propagation has been observed in layer II of the ERC in middle-aged rhesus monkeys and in layer III of dlPFC in older animals (Paspalas et al., 2018; Datta et al., 2024; Carlyle et al., 2014). Similar tau “seeding” behavior has been documented in human sAD brain tissue, where the ERC demonstrated the highest efficiency in transmitting tau pathology (Kaufman et al., 2018). Mouse models have also provided evidence for tau propagation between neurons (Gibbons et al., 2019). However, the rhesus monkey studies offer direct visualization of this process at the nanoscale and suggest a plausible mechanism by which tau pathology may spread through association cortical circuits at very early stages of the disease, providing a remarkable opportunity to understand how this intersects with other pathological sequelae, e.g., inflammatory cascades (see below).

In human AD, affected neurons ultimately succumb to autophagic degeneration, leaving behind characteristic “ghost tangles” [reviewed in Arnsten et al. (2021)]. Correspondingly, our research has identified autophagic vacuolar degeneration in dendrites of aged rhesus macaques—specifically in layer II of the ERC in “early-aged” animals, and in layer III of the dlPFC in “late-aged” animals—mimicking the degenerative trajectory observed in human sAD (Paspalas et al., 2018; Datta et al., 2024). Strikingly, extremely aged rhesus macaques develop classic NFTs in the ERC, composed of paired helical filaments identical in size and helical frequency to those found in human sAD, and immunolabeled by the diagnostic AT8 antibody (pS202/pT205Tau) (Paspalas et al., 2018). These animals also show significant impairments in recognition memory (Paspalas et al., 2018), paralleling early cognitive symptoms in humans.

Age-related structural changes in rhesus macaques also align with human pathology. Notably, there are selective decreases in dendritic spine density in the dlPFC, but not in V1 (Young et al., 2014), consistent with the pattern of synaptic loss seen in human AD (DeKosky and Scheff, 1990). In addition, aged rhesus macaques naturally develop amyloid plaques with similar morphology and dimensions to those in humans (Uno and Walker, 1993; Mufson et al., 1994). Beyond amyloid and tau pathology, these monkeys also display a range of AD-like neurodegenerative features, including mitochondrial dysfunction, inflammatory pathway activation, microglial engulfment of synapses, synaptic degeneration, argyrophilic deposits, buildup of late-phase lysosomes, and dystrophic neurites (Paspalas et al., 2018; Datta et al., 2024; Datta and Arnsten, 2025). Rhesus macaques of extreme age also exhibit pronounced recognition memory deficits as well as impaired executive functions (Arnsten et al., 2021; Rapp and Amaral, 1989). These pathological similarities to human sAD provide construct validity in using non-human primate models to investigate the etiology of inflammatory dysfunction in neurodegenerative diseases.

## Strengths and limitations of rodent and non-human primate models in AD research

While non-human primate (NHP) models offer unique advantages for studying the earliest etiological events of sAD—particularly those involving higher-order association cortices, primate-specific immune signaling, and soluble tau species—they also present practical and conceptual limitations (Defelipe, 2011; Hardingham et al., 2018; Hill and Walsh, 2005; Preuss, 2012; Sousa et al., 2017). NHP studies are resource-intensive, have lower throughput, and offer limited opportunities for large-scale genetic manipulation, which can constrain mechanistic dissection of complex gene–gene and gene–environment interactions (Defelipe, 2011; Hardingham et al., 2018; Hill and Walsh, 2005; Preuss, 2012; Sousa et al., 2017). In contrast, mouse models provide unparalleled genetic tractability, enabling precise manipulation of individual inflammatory, metabolic, and synaptic pathways and rapid testing of causal relationships. Rodent systems are therefore especially powerful for probing multifactorial genetic risk, screening therapeutic targets, and defining molecular cascades underlying neuroinflammation. However, key aspects of sAD—including association cortex architecture, microglial transcriptional states, neuroinflammatory mechanisms, neuromodulatory control of calcium signaling, and the emergence of soluble tau pathology—are either absent or fundamentally different in rodents. Accordingly, we view rodent and NHP models as complementary rather than competing systems: mouse models are ideally suited for mechanistic discovery and hypothesis generation, whereas aging NHPs are essential for validating disease-relevant mechanisms and therapeutic strategies in a translationally faithful neuroimmune and cortical context.

## Gradients in intracellular Ca^2+^ signaling and inflammation across the cortical hierarchy and evolution

**Figure 1:**
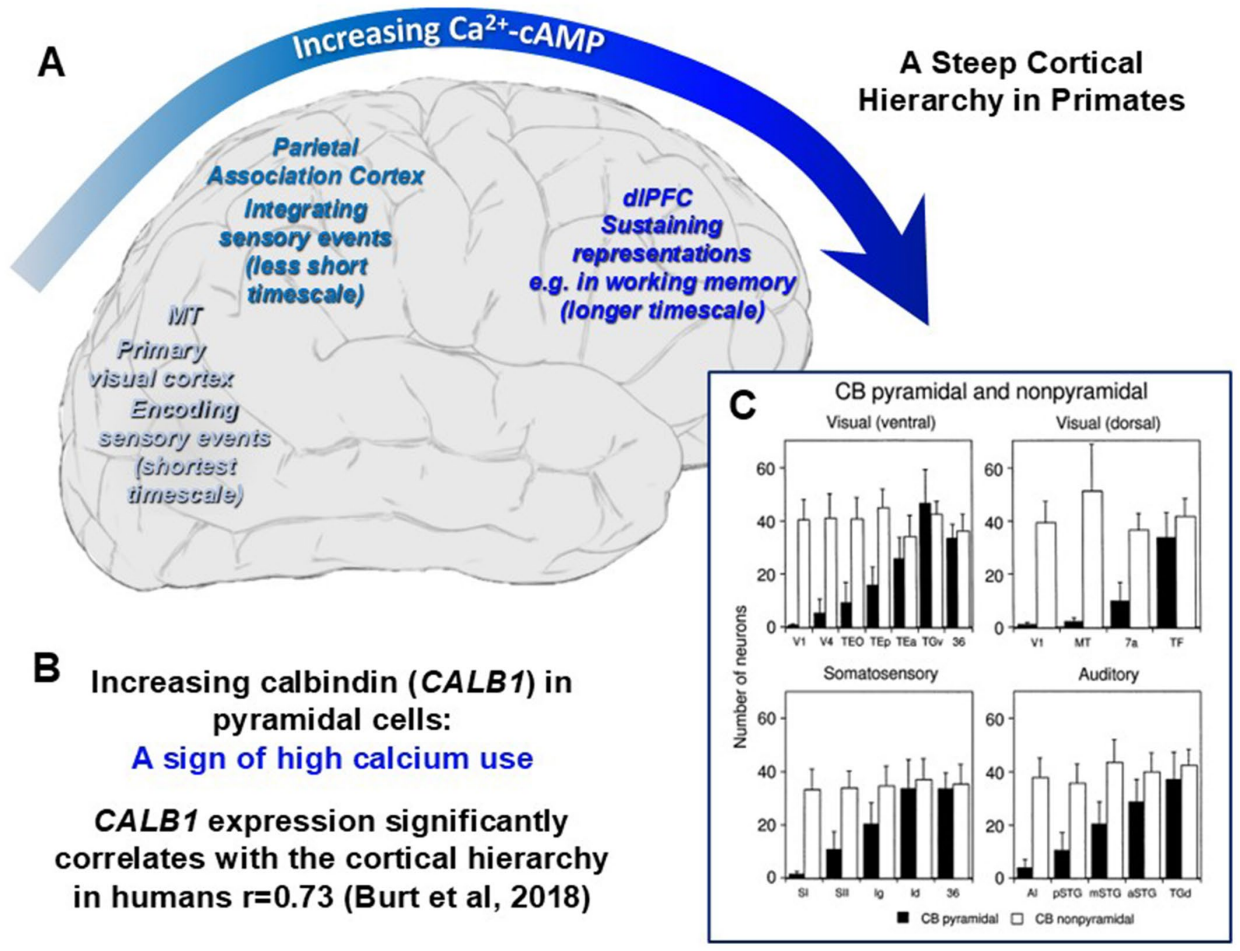
Amplification of Ca^2+^–cAMP signaling across the primate cortical hierarchy. **(A)** Schematic of the human cortical hierarchy showing progressively longer intrinsic timescales, from the shortest in primary visual cortex (V1) and early visual area MT, to progressively longer ones in association and limbic cortices (LIP/7a = parietal association cortices; dlPFC = dorsolateral prefrontal cortex). Timescale data from Murray et al. (2014). Furthermore, dendritic spine density on layer 3 pyramidal neurons increases both across the cortical hierarchy and throughout primate evolution (Elston, 2000; Elston, 2003; Elston et al., 2006). (**B)** Expression of the Ca^2+^-binding protein calbindin (*CALB1*) rises along the human cortical hierarchy (Burt et al., 2018). (**C)** Histology data in macaque cortex shows calbindin expression rises along the cortical hierarchy in a cell-type specific manner in pyramidal cells but not in interneurons (Kondo et al., 1999). CB, calbindin.

The primate cortex exhibits a steep hierarchical, lattice-like organization (Magrou et al., 2024). Distinct functional and structural differences emerge as one moves from the primary sensory areas through sensory association regions, up to higher-order cognitive association cortices, and to limbic cortices involved with emotion. As schematically illustrated in Figure 1A, computational studies have shown that neurons across this hierarchy operate on progressively longer timescales, meaning that a neuron’s current activity is increasingly shaped by its past activity—supporting processes like sensory integration, working memory, and sustained emotional states (Murray et al., 2014; Arnsten et al., 2021; Monosov et al., 2020).

These functional gradients are mirrored by anatomical and molecular features. Pyramidal neurons in higher-order regions exhibit greater dendritic spine density than those in lower regions such as primary visual cortex [V1; Elston et al. (2001) and Elston et al. (2011)], and increased expression of calcium-related genes, including *GRIN2B* (which encodes the slow-closing, high-calcium-flux NMDA receptor subunit GluN2B), and *CALB1* (calbindin), a marker of elevated intracellular calcium handling (Figure 1B) (Burt et al., 2018). In macaques, hierarchical expression of calbindin is driven by pyramidal neurons, not interneurons, consistent with their expanding synaptic inputs across the cortical hierarchy (Figure 1C) (Kondo et al., 1999), including further expansion of calbindin expression in pyramidal cells of the limbic cortices (Joyce et al., 2020). Calcium signaling is often increased by cAMP-PKA signaling (Arige and Yule, 2022), and proteomic data from human brain shows increasing expression of *PDE4D* and *GRM3* (mGluR3)—both key regulators of cAMP–PKA signaling—along the hierarchy, with the highest levels in the dlPFC and lowest in V1 (Carlyle et al., 2017). Similarly, there is an expansion of stress-related genes in layer III pyramidal cells across the cortical hierarchy, including increases in dopamine D1 receptors (*DRD1*) and the gene encoding the “master stress peptide,” PACAP (Pituitary Adenylate Cyclase-Activating Polypeptide) (Bian et al., 2024), both of which increase cAMP and/or calcium signaling. There is also a hierarchical increase in the gene encoding the SK3 potassium channel (Enwright et al., 2022; Arion et al., 2023), which is opened by calcium, and causes calcium to reduce neuronal firing in the dlPFC (Datta et al., 2024). Inflammatory pathways and cell-types also show gradients across cortical hierarchy. Transcriptomic profiling across 15 brain regions in rhesus macaques shows regionally graded age-associated gene expression changes, especially in immune and neurodegeneration-related genes, particularly affecting association cortices like the dlPFC (Chiou et al., 2022). Notably, the pattern of calbindin expression across pyramidal neurons aligns strikingly with the regions most affected by tau pathology and neurodegeneration in AD (Arnsten et al., 2021).

Interestingly, this hierarchical pattern for calcium signaling and inflammatory mechanisms has also expanded during primate evolution. In humans, there is a marked increase in both dendritic spine density and *GRIN2B* expression compared to simpler primates (Elston et al., 2001, 2011; Muntané et al., 2015). These species-specific features are highly relevant to AD, as many neuroinflammatory processes show expanded or altered expression in humans relative to rodents (Kodamullil et al., 2017), and mice have a less differentiated cortical hierarchy (Magrou et al., 2024; Gilman et al., 2017). For example, microglia in rodents typically exhibit pronounced state transitions, cytokine levels, and morphological changes following acute insults; whereas microglia in primates exhibit more gradual, region-specific alterations with more subtle microglial process velocity, dystrophic morphologies, and chronic low-level cytokine increases during aging [reviewed in Edler et al. (2021)]. In addition, cross-species single-cell analysis reveals distinct microglial gene modules in primates, including unique complement pathway and inflammatory-related expression patterns not seen in rodents (Geirsdottir et al., 2019). As described below, inflammatory pathways affecting NMDAR neurotransmission and mGluR3 regulation of cAMP-calcium signaling are also expanded in primates. Thus, primate models are especially valuable for studying early disease mechanisms that are absent or poorly represented in rodent brains—particularly in excitatory neurons and glial cell-types in higher-order cortical regions.

## Magnified calcium signaling in higher cortical glutamate synapses needed for cognition

**Figure 2:**
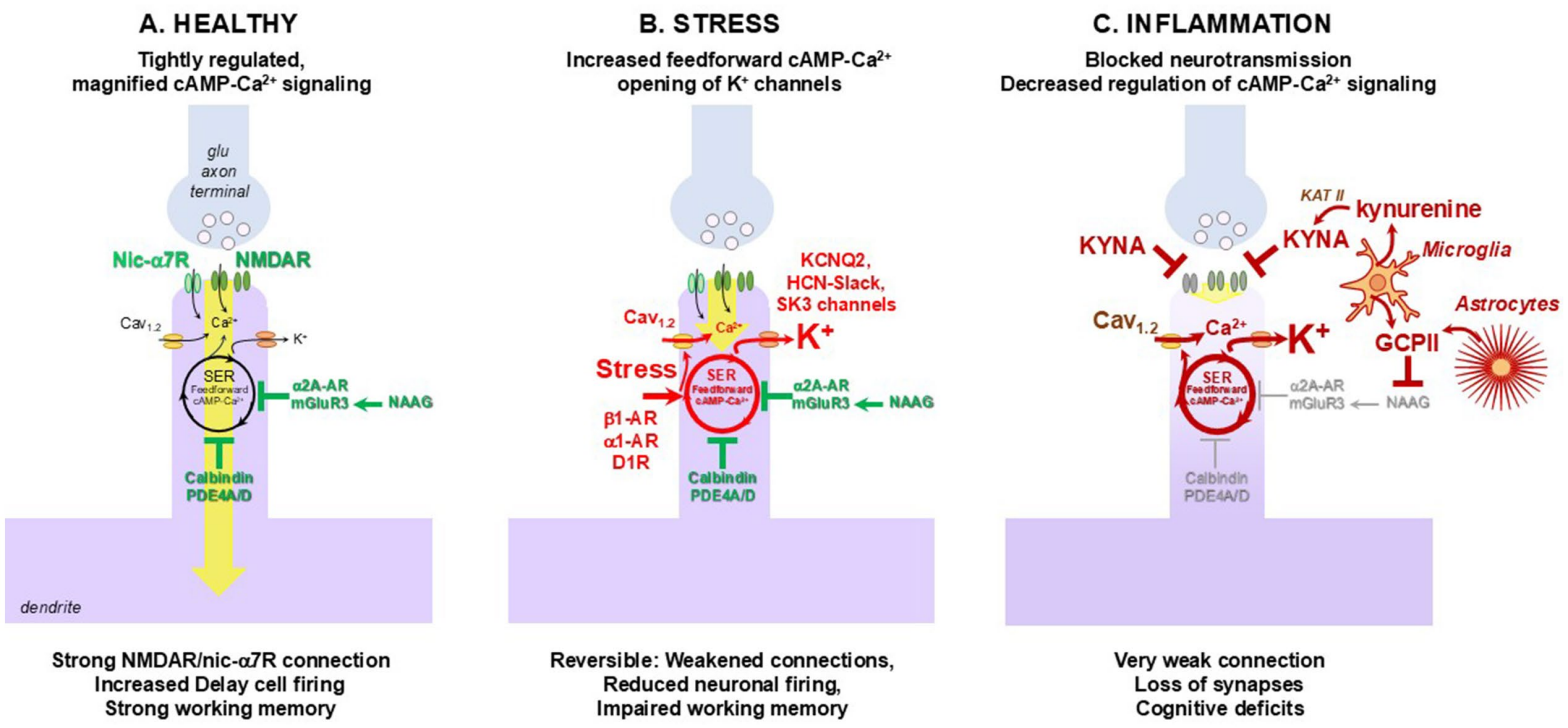
Schematic of glutamatergic synapses in excitatory microcircuits in primates mediating higher-order cognition and susceptibility to stress and inflammation. **(A)** Illustration of a glutamatergic synapse on a dendritic spine in the young, healthy association cortex, characterized by tightly regulated feedforward Ca^2+^–cAMP–K^+^ channel signaling. Neurotransmission in these circuits depends on NMDARs containing GluN2A and GluN2B subunits, with permissive modulation by cholinergic Nic-α7R and M1R (the latter acting via inhibition of KCNQ5 channels, not shown). Calcium influx via LVCC Cav1.2 channels on dendritic spines in peri- and extra-synaptic compartments are also critical for neuronal firing. Spines contain molecular machinery that amplifies Ca^2+^ signaling required for persistent firing, including cAMP–PKA–mediated facilitation of internal Ca^2+^ release from the smooth endoplasmic reticulum (SER) spine apparatus. This Ca^2+^ release further enhances cAMP production, generating feedforward cAMP–Ca^2+^ signaling. Dendritic spines also express K^+^ channels (e.g., HCN-Slack, KCNQ2, SK3) activated by cAMP–PKA-Ca^2+^ signaling, providing negative feedback and enabling dynamic modulation of network connectivity. Under physiological conditions, these intracellular pathways are strictly controlled by phosphodiesterase type 4 (PDE4) enzymes anchored to the SER by DISC1 that degrade cAMP, and the Ca^2+^-binding protein calbindin. PDE4s are also positioned in dendrites near mitochondria to regulate cAMP-driven Ca^2+^ transfer from the SER to mitochondria (not shown). Feedforward calcium–cAMP signaling is tightly regulated by the G_i/o_-coupled receptors mGluR3 and α2A-AR, which are localized on dendritic spines and suppress cAMP synthesis. mGluR3 receptors are activated not only by glutamate but also by *N*-acetylaspartylglutamate (NAAG), which is co-released with glutamate and acts selectively on mGluR3. Under healthy conditions, NAAG stimulation of mGluR3 on dendritic spines enhances neuronal firing by inhibiting cAMP–PKA–mediated opening of K^+^ channels. Note, much of the physiological, molecular and functional characterization of primate glutamatergic circuits has been conducted in macaque dlPFC, but similar signatures have been observed in ERC circuits. **(B)** Acute exposure to uncontrollable stress triggers elevated catecholamine release in the excitatory circuits in the PFC, activating feedforward cAMP–Ca^2+^–K^+^ channel signaling that rapidly weakens synaptic efficacy, reduces persistent neuronal firing, and functionally takes the PFC “offline,” e.g., dlPFC that is essential for top-down control. Multiple receptors localized to dendritic spines engage this pathway, including dopamine D1R, and norepinephrine α1-adrenoceptor (α1-AR) and β1-adrenoceptor (β1-AR). Cortisol release further amplifies—or independently reproduces—these effects, likely by inhibiting extraneuronal catecholamine transporters on glia that normally clear catecholamines from the extrasynaptic space. Regulation, e.g., by PDE4s, would allow connectivity to return to normal once the stress is over. **(C)** With chronic stress and/or inflammation, regulation of cAMP-Ca^2+^ signaling is lost, and chronic weakening leads to atrophy of spines and dendrites that correlate with impairments in cognitive performance. Although calcium dysregulation can activate inflammatory cascades, the reverse is also true—neuroinflammation can disrupt cAMP–calcium signaling, creating a self-reinforcing cycle that promotes neuronal atrophy. Inflammatory signaling can induce multiple molecular alterations that impair higher-order function and mirror the effects of genetic vulnerabilities. For instance, activation of the MK2 inflammatory pathway leads to inactivation and disanchoring of PDE4, preventing its proper localization to sites where it normally restrains cAMP-driven calcium release. The resulting rise in cytosolic calcium is especially harmful when the calcium-buffering protein calbindin—lost from pyramidal neurons but preserved in interneurons during aging—is reduced, thereby impairing calcium homeostasis within intracellular compartments. Importantly, inflammation also elevates expression of molecules that diminish network connectivity and neuronal firing in glutamatergic circuits in higher-order association cortices, including glutamate carboxypeptidase II (GCPII) and kynurenic acid (KYNA). Astrocytes and microglia synthesize the enzyme for GCPII, which catabolizes NAAG and thereby reduces mGluR3-mediated signaling. Under inflammatory conditions, glial cell-types, both microglia and astrocytes, increase GCPII synthesis and release, decreasing mGluR3 regulation of intracellular calcium within postsynaptic compartments in higher-order glutamatergic circuits. Thus, increases in GCPII expression with inflammation contribute to cognitive deficits in aging and sAD. Similarly, under inflammatory conditions, microglia metabolize tryptophan to kynurenine, which can be further metabolized to KYNA. KYNA blocks nicotinic-a7R as well as NMDAR, the two receptors most needed for dlPFC neurotransmission, and KYNA markedly reduces the dlPFC delay cell firing in macaque dlPFC needed for working memory. cAMP, cyclic adenosine monophosphate; Nic-α7R, nicotinic α7 receptor; NMDAR, NMDA receptor; PDE4, phosphodiesterase type 4; SER, smooth endoplasmic reticulum; LVCC, L-type voltage-gated calcium channel.

**Figure 3:**
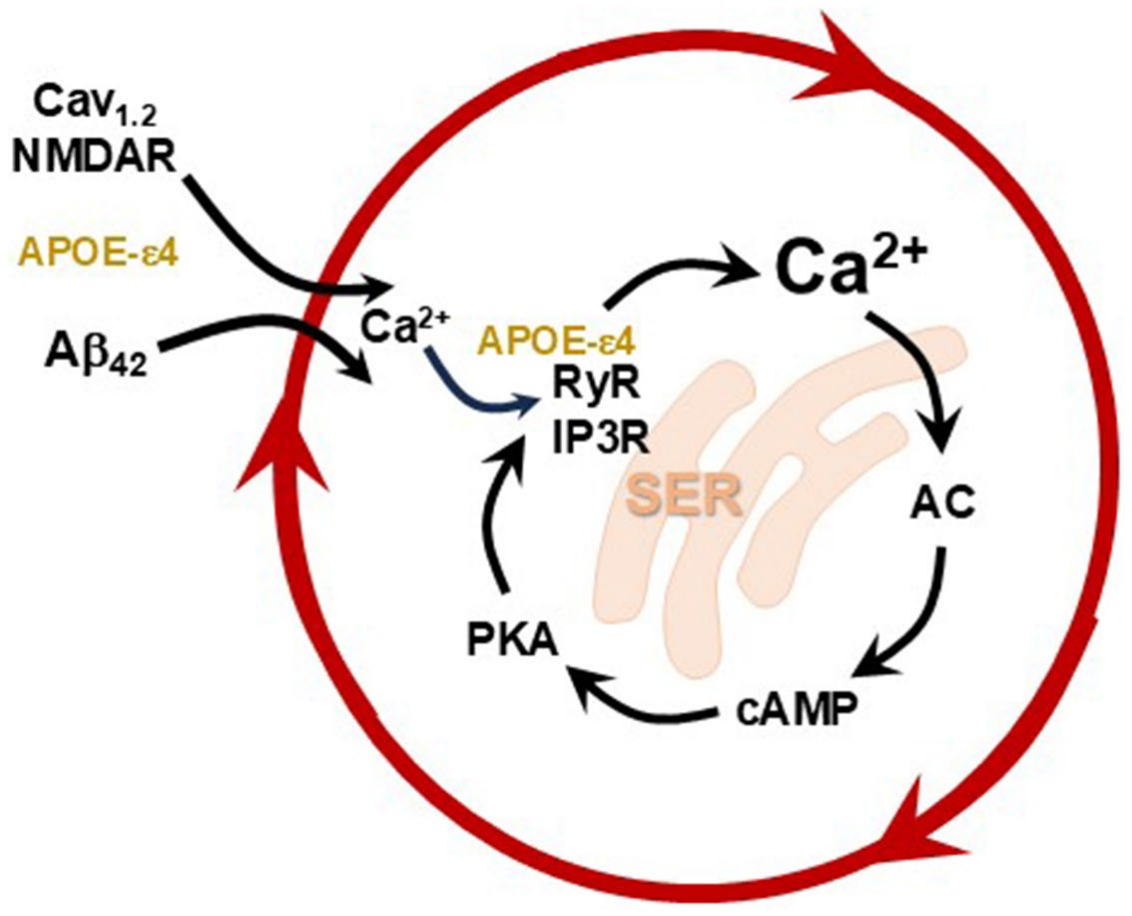
A schematic diagram illustrating how L-type voltage gated calcium channels (L-VGCCs) Cav1.2, NMDARs, and Aβ₄₂ drive Ca^2+^ dysregulation, exacerbated by APOE-e4 genotype. The smooth endoplasmic reticulum (SER) is a key nexus of pathology, as calcium dysregulation further exacerbates calcium release from RyR and IP3R calcium channels on the SER. Cytosolic Ca^2+^ can in turn activate adenylyl cyclase (AC) to increase cAMP and PKA production, thus creating feedforward signaling. The APOE-ε4 genotype amplifies multiple facets of intracellular Ca^2+^ dysregulation and inflammatory signaling (Wang et al., 2022). For instance, APOE influences intraneuronal free Ca^2+^ levels in a dose-dependent manner—APOE-ε4 producing the highest levels, followed by APOE-ε3, and then APOE-ε2—mirroring their relative contributions to sAD risk (Ohm et al., 2001). APOE-ε4 produces a prolonged elevation of intracellular Ca^2+^ by stimulating both NMDARs and L-VGCCs (Ohkubo et al., 2001; Ramakrishna et al., 2021). APOE-ε4 further enhances Ca^2+^ release from the SER through ryanodine receptor activation (Ohkubo et al., 2001).

The sustained firing of dlPFC neurons is needed to maintain information in working memory across a delay when sensory stimulation is no longer available. These dlPFC “Delay cells” are thought to reside in layer III, and exhibit unusual neurotransmission and neuromodulation required for flexible but sustained neuronal firing. dlPFC Delay cells rely heavily on NMDAR neurotransmission (Figure 2A), including receptors with GluN2B as well as GluN2A subunits within the post-synaptic density (PSD). As mentioned above, NMDARs containing GluN2B conduct particularly large calcium currents. NMDAR neurotransmission can only occur when the PSD is depolarized, ejecting Mg^2+^ from the NMDAR ion pore (Nowak et al., 1984). In a typical glutamate synapse, glutamate stimulation of AMPARs supply these permissive excitatory actions (Bekkers and Stevens, 1989). However, for layer III dlPFC Delay cells, the permissive excitation is supplied by acetylcholine, including by nicotinic a7-receptors (nic-a7R) in the glutamate PSD (Figure 2A) (Yang et al., 2013). Nic-a7R also flux calcium into the cell, which may be helpful in maintaining a depolarized PSD (Yang et al., 2013). Calcium is also contributed by L-type voltage-gated channels such as Cav_1.2_ (Datta et al., 2024), and by internal calcium release from the smooth endoplasmic reticulum (SER), which is called the spine apparatus within dendritic spines (Figure 2A) (Datta et al., 2024). cAMP-PKA signaling drives calcium release from the SER and through Cav_1.2_, and calcium in turn drives more cAMP production, thus creating feedforward cAMP-calcium signaling (seen in more detail in Figure 3). Under healthy conditions, feedforward cAMP-calcium signaling in layer III dlPFC pyramidal cells is tightly regulated by calbindin buffering of calcium, phosphodiesterase (PDE4) catabolism of cAMP, and α2A-AR and mGluR3 inhibition of cAMP production (Figure 2A). Unlike in rodents where mGluR3 are predominately presynaptic, in primate layer III dlPFC they are post-synaptic on dendritic spines, where they inhibit cAMP drive on calcium release (Jin et al., 2018; Jin et al., 2017). mGluR3 are stimulated not only by glutamate, but by NAAG, which is co-released with glutamate and is selective for mGluR3 (Figure 2A). NAAG greatly increases dlPFC Delay cell firing, emphasizing the power of this mechanism in primates (Jin et al., 2018; Jin et al., 2017).

Under conditions of uncontrollable stress, high levels of catecholamine release in the dlPFC drives feedforward cAMP-calcium signaling to open nearby K^+^ channels to rapidly weaken network connections and reduce Delay cell firing (Figure 2B) (Datta and Arnsten, 2019; Woo et al., 2021; Datta and Arnsten, 2018). High levels of norepinephrine release during stress engage low affinity, α1-AR and β1-AR; the latter activate nearby Cav_1.2_ currents, similar to stress actions in heart (Datta et al., 2024; Datta et al., 2019). High levels of cytosolic calcium in turn open SK channels, PKA opens KCNQ2 channels, and cAMP opens HCN-Slack channels on spines, reducing the efficacy of NMDAR synapses, decreasing Delay cell firing and switching control of behavior to more primitive circuits that are strengthened by high levels of norepinephrine release (Datta and Arnsten, 2019; Ramos et al., 2003; Vijayraghavan et al., 2007). In healthy subjects, regulation, e.g., by PDE4s, restores dlPFC connectivity, and these rapid changes in network connectivity are a “signature of flexibility” that promote coordination of arousal state with cognitive state, a mechanism termed Dynamic Network Connectivity (Arnsten et al., 2012; Arnsten et al., 2022). However, with chronic stress and/or inflammation, PFC connections are lost. In this context, it is noteworthy that stress is a risk factor for future AD (Datta and Arnsten, 2025; Arnsten et al., 2019; Arnsten and Datta, 2024; Arnsten et al., 2025; Bathla et al., 2023).

It is not known whether this “signature of flexibility” is common to other association cortical synapses, but it is highly relevant to the etiology of AD that layer II of the ERC, which are the first cortical cells to show tau pathology, exhibit this signature on spines and at excitatory synapses on dendrites of excitatory neurons (Datta et al., 2023). However, the layer II cell islands in ERC have sparse expression for calbindin, even when young and healthy (Beall and Lewis, 1992). The expression of magnified calcium signaling in the absence of protective factors may contribute to vulnerability to tau pathology.

## Inflammation weakens higher cortical connections needed for cognition

Inflammation weakens layer III dlPFC connections on dendritic spines by blocking neurotransmission, and by dysregulating the stress response (Figure 2C). Under inflammatory conditions, microglia metabolize tryptophan to kynurenine, which can be further metabolized to kynurenic acid (KYNA; Figure 2C) or quinolinic acid (Badawy, 2017). Kynurenine is also generated by the peripheral immune system and is actively taken up from blood into brain (Stone and Williams, 2023). Much of the research in this field has focused on the excitotoxic effects of quinolinic acid, which stimulates NMDAR, with KYNA referred to as the protective metabolite, as it blocks NMDAR (Stone and Addae, 2002). Although this may be true under conditions of excess glutamate, such as during stroke, KYNA’s actions may be detrimental to cognition under conditions of normal or reduced glutamate actions. It is noteworthy that KYNA blocks nicotinic-a7R as well as NMDAR, the two receptors most needed for dlPFC neurotransmission (Albuquerque and Schwarcz, 2013). Recent research shows that KYNA markedly reduces the dlPFC Delay cell firing in macaque dlPFC needed for working memory (Figure 2C), and conversely, that inhibiting the production of KYNA can restore neuronal firing and working memory performance in aged macaques (Yang et al., 2024). Thus, elevated KYNA in dlPFC is detrimental to higher cognitive function, and this is especially true in primates. KYNA is metabolized from kynurenine by KAT II, and transcriptomic analyses show a great expansion in the gene encoding KAT II from mice to primates, including extensive expression within neurons in macaque and human dlPFC (Yang et al., 2024). This may reflect the parallel expansion of NMDAR-GluN2B expression in primate dlPFC (Muntané et al., 2015), and helps to explain why so many neuroinflammatory disorders, e.g., long-COVID, are associated with dlPFC cognitive impairment (Vanderlind et al., 2021). Interestingly, KYNA activates IDO, the enzyme that metabolizes tryptophan to kynurenine, and thus sustains its own production (Badawy, 2023). This may contribute to the prolonged nature of many neuroinflammatory disorders, such as long-COVID (Cysique et al., 2023). KYNA is elevated in the early stages of AD (Almulla et al., 2022), and increased plasma kynurenine correlates with measures of Aβ and neurofilament light chain assays of degeneration (Chatterjee et al., 2019). It is noteworthy that sustained exposure to KYNA *in vitro* causes synapse loss (Orhan et al., 2025), and disorders associated with elevated KYNA such as schizophrenia (Schwarcz et al., 2001; Kindler et al., 2020) and AD (Widner et al., 2000) are associated with synapse loss in the dlPFC (DeKosky and Scheff, 1990; Glantz and Lewis, 2000).

Advanced age and/or inflammation also causes dysregulation of stress response in primate dlPFC, with loss of calbindin, PDE4A and PDE4D and reduced mGluR3 regulation of cAMP-calcium signaling (Figure 2C) (Datta and Arnsten, 2025). Inflammation induces microglia and astrocytes to generate and release GCPII (glutamate carboxypeptidase II) which catabolizes NAAG, the endogenous ligand for mGluR3, thus dysregulating feedforward cAMP-calcium-K^+^ channel signaling (Figure 2C) (Datta and Arnsten, 2025). Conversely, inhibiting GCPII greatly enhances Delay cell firing and improves working memory in aged macaques (Yang et al., 2022). GCPII activity is also related to tau pathology, and GCPII inhibitors may have therapeutic potential.

**Figure 4:**
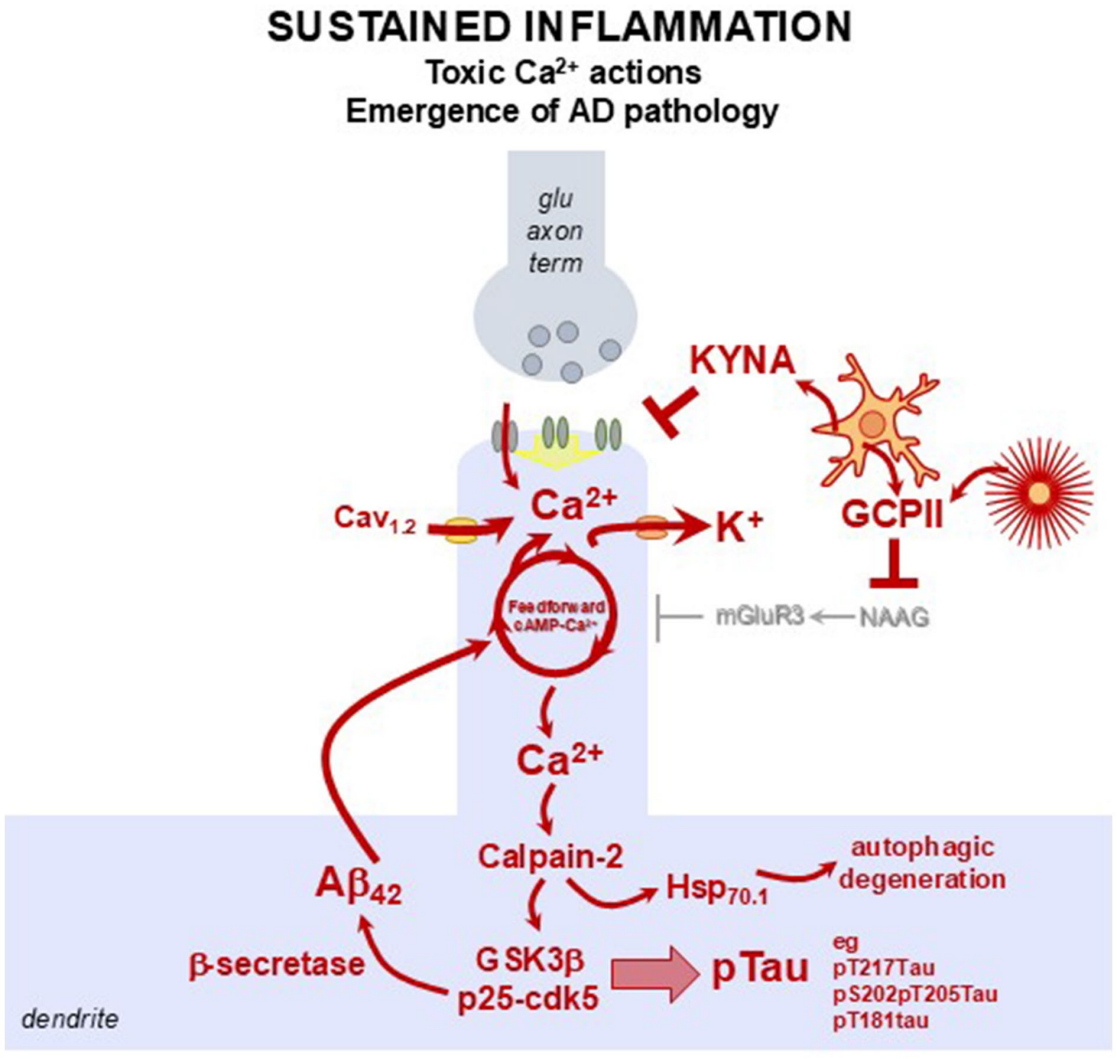
Sustained inflammation and/or abrogated cAMP–Ca^2+^ regulatory mechanisms with aging disrupts neuronal firing and promotes AD pathology. A schematic illustrating how age- or inflammation-related reductions in calbindin, PDE4 enzymes, and α2A-AR/mGluR3 modulation destabilize cAMP–Ca^2+^ signaling, leading to K^+^ channel opening, reduced dlPFC neuronal firing, and AD pathology. When cytosolic Ca^2+^ rises sufficiently to activate calpain-2, a cascade of toxic events follows: calpain-2 cleaves and disinhibits GSK3β, converts p35–cdk5 to the pathogenic p25–cdk5 form that hyperphosphorylates tau at multiple epitopes (e.g., pT217Tau, pS202/T205Tau, pT181Tau), and cleaves heat shock protein 70.1 (hsp70.1), triggering lysosomal dysfunction and autophagic degeneration. Activation of p25–cdk5 also enhances β-secretase processing of APP, increasing production of Aβ_42_. Aβ_42_ further increases Ca^2+^ levels, creating vicious cycles. Thus, dysregulation of feedforward calcium–cAMP signaling promotes excessive activation of nearby K^+^ channels, weakening synaptic connectivity, driving spine loss, and reducing the persistent firing required for higher-order cognitive function. Over a lifetime, sustained elevations in intracellular calcium may exert multiple neurotoxic effects, including enhanced tau phosphorylation (pTau), increased amyloid deposition, neuroinflammation, and ultimately, neurodegeneration.

With sustained inflammation, dysregulated cAMP-calcium signaling leads to tau and amyloid pathology and autophagic degeneration, as summarized in Figures 3, 4 [reviewed in Datta and Arnsten (2025), Arnsten et al. (2019), Arnsten et al. (2021), and Arnsten et al. (2025)]. Very high levels of cytosolic calcium activate calpain-2, which cleaves and disinhibits a number of culprits known to drive AD pathology. Calpain-2 cleaves off the regulatory end of GSK3β, a major kinase involved with tau hyperphosphorylation, and it cleaves p35 to p25, which activates cdk5, another key kinase in tau hyperphosphorylation. Activated cdk5 also increases β-secretase cleavage of APP to Aβ_42_, thus increasing amyloid pathology. Ab_42_ and the AICD peptide cleaved from APP both increase internal calcium release, and Ab_42_ can create calcium pores in the plasma membrane, further driving pathology. Calpain-2 also cleaves and activates Hsp_70.1_ which drives autophagic degeneration, the manner by which neurons die in AD. Thus, inflammation can drive multiple aspects of AD pathology via calcium dysregulation in dlPFC, and in other vulnerable neurons.

## Calcium and inflammation pathways as a nexus for sporadic AD risk in association cortices

### Key calcium-related genetic risk factors in Alzheimer’s disease

As described, longstanding research has shown that calcium dysregulation in aging association cortices plays a central role in the development of tau pathology in sAD (Khachaturian, 1991; Mattson, 2007; Stutzmann, 2007; Gibson and Thakkar, 2017; Area-Gomez and Schon, 2017). Our working hypothesis posits that pyramidal neurons in higher-order association cortices are especially reliant on tightly regulated intracellular calcium signaling to support complex cognitive functions like working memory, attention, and executive functions (Arnsten et al., 2021; Datta and Arnsten, 2025; Arnsten et al., 2022). With advanced age, multiple factors, including chronic inflammation, genetic predispositions, and environmental exposures, converge to destabilize calcium homeostasis (Figure 4). This disruption can lead to a cascade of detrimental processes including tau hyperphosphorylation, synaptic dysfunction, and eventual neurodegeneration (Arnsten et al., 2021; Datta and Arnsten, 2025; Arnsten et al., 2022). Understanding how calcium and inflammatory signaling intersects offer critical insight into the earliest etiological events in sAD and highlight new avenues for preventive therapy.

A number of genetic risk factors for sporadic AD directly impact calcium regulation. The APOE ε4 allele, the strongest known genetic risk factor for sAD, has been shown to impair mitochondrial function and endolysosomal trafficking, and promote amyloidosis (Holtzman et al., 2012; Raulin et al., 2022; Corder et al., 1994; Strittmatter et al., 1993; Martens et al., 2022; Zalocusky et al., 2021; Gonneaud et al., 2016; Mishra et al., 2018; Murphy et al., 2013; Liu et al., 2017). These disruptions interfere with neuronal calcium buffering capacity, leading to elevated cytosolic calcium levels (Serrano-Pozo et al., 2015; Morrison et al., 2024). As schematically illustrated in Figure 3, APOE ε4 also increases intracellular calcium levels by activating NMDARs and L-type voltage-gated Ca2 + channels (Ohkubo et al., 2001; Ramakrishna et al., 2021), and by increasing calcium release from the SER via ryanodine receptors (Ohkubo et al., 2001). It also impairs calcium handling by lysosomes which can contribute to degeneration (Larramona-Arcas et al., 2020). APOE ε4 also increases susceptibility to calcium overload, making neurons more vulnerable to excitotoxic damage in response to inflammatory and metabolic stress (Pires and Rego, 2023).

Although mutations in presenilin-1 (*PSEN1*) are classically associated with familial AD, emerging evidence suggests that some PSEN1 variants also modulate calcium signaling in sAD (Khachaturian, 1991; Mattson, 2007; Stutzmann, 2007; Gibson and Thakkar, 2017; Area-Gomez and Schon, 2017). PSEN1 plays a role in controlling calcium leak in the endoplasmic reticulum (ER), and dysfunction in these calcium channels results in excessive release of calcium into the cytoplasm (Khachaturian, 1991; Mattson, 2007; Stutzmann, 2007; Gibson and Thakkar, 2017; Area-Gomez and Schon, 2017). Even without amyloidogenic mutations, these variants can elevate baseline intracellular calcium levels and sensitize neurons to further dysregulation, promoting tau hyperphosphorylation and early synaptic loss. *CALHM1* (calcium homeostasis modulator 1) encodes a membrane channel involved in calcium influx and ATP release, both of which are important for maintaining neuronal excitability and intercellular communication. Polymorphisms in CALHM1 have been linked to increased production of Aβ, possibly through calcium-dependent regulation of APP cleavage (Dreses-Werringloer et al., 2008). These variants also disrupt calcium handling within neurons, leading to prolonged calcium elevations that stress intracellular signaling pathways and contribute to pathology. Another important risk gene is *CACNA1C*, which encodes the alpha-1C subunit of L-type voltage-gated calcium channels (Cav1.2). These channels are highly expressed in association cortices, and regulate calcium entry into dendrites and spines during synaptic activity (Datta et al., 2024). Genetic variants in *CACNA1C* are associated with altered cognitive function, increased risk for AD, and psychiatric disorders such as bipolar disorder and schizophrenia. Overactivation of Cav1.2 channels can lead to excessive calcium influx, which in turn activates kinases such as CaMKII and GSK3β, key drivers of tau phosphorylation (Ekinci et al., 1999; Thibault et al., 2001; Thibault and Landfield, 1996; Ueda et al., 1997; Willis et al., 2010). This mechanism may help explain the shared circuit vulnerability seen across neurodegenerative and neuropsychiatric conditions. Finally, calcium release from intracellular stores is also governed by receptors such as *ITPR2* (inositol 1,4,5-trisphosphate receptor type 2) and *RYR2* (ryanodine receptor type 2), which are located on the ER membrane. These receptors respond to intracellular signaling molecules like IP3 and cAMP, mediating rapid calcium release into the cytoplasm. With aging and inflammatory stress, these receptors can become dysregulated, leading to sustained or exaggerated calcium signaling (Bruno et al., 2012; Chakroborty et al., 2009; Cheung et al., 2010; Goussakov et al., 2010; Kelliher et al., 1999; Stutzmann, 2005; Stutzmann et al., 2004; Zhang et al., 2023). Genetic variations in *ITPR2* and *RYR2* have been linked to altered calcium dynamics in aging neurons and may contribute to their selective vulnerability in AD (Bruno et al., 2012; Chakroborty et al., 2009; Cheung et al., 2010; Goussakov et al., 2010; Kelliher et al., 1999; Stutzmann, 2005; Stutzmann et al., 2004; Zhang et al., 2023). Furthermore, aging and inflammation increase oxidative and nitrosative stress, which can modify RyR2 through redox-dependent mechanisms, destabilizing the channel and further enhancing calcium leak independently of, and in combination with, PKA-mediated phosphorylation (Cooper et al., 2013; Leyane et al., 2022; Nikolaienko et al., 2018; Giorgi et al., 2018). These oxidative mechanisms may act within confined dendritic nanodomains to exacerbate calcium dysregulation and amplify vulnerability to tau phosphorylation.

In summary, multiple genetic factors implicated in sAD converge on calcium signaling pathways, particularly in the association cortices that support higher cognition. These regions are not only structurally complex and energetically demanding but also highly sensitive to the effects of aging and inflammation. Disruptions in calcium homeostasis—whether through impaired buffering, excessive calcium influx, or abnormal calcium release from intracellular stores—create a permissive environment for tau pathology and Aβ accumulation. Because many of these calcium-related mechanisms are modifiable, they represent promising targets for early intervention, particularly in individuals with heightened genetic risk such as APOE ε4 carriers. In particular, the natural ApoE ε4/ε4 status of rhesus macaques may bias the model toward mechanisms that are particularly relevant to ApoE4 carriers, while also providing unique insight into sAD pathophysiology.

### Key inflammation-related genetic risk factors in Alzheimer’s disease

A central theme emerging in AD research is the pivotal role of chronic neuroinflammation in the onset and progression of the disease (Bettcher et al., 2021; Haage and De Jager, 2022). Several genetic risk factors for sAD influence the immune system’s ability to regulate inflammatory responses in the brain, particularly through the function of microglia—the resident immune cells of the central nervous system (Bettcher et al., 2021; Haage and De Jager, 2022; Gao et al., 2023). These genetic variants can either exacerbate or impair microglial responses, thereby affecting Aβ and pathological tau clearance, synaptic integrity, and overall neuronal health.

Various inflammation related risk factors have been implicated in the pathogenesis of sAD. For example, the APOE ε4 allele exerts powerful pro-inflammatory effects in the brain (Shi et al., 2019; Yin et al., 2023; Rosenzweig et al., 2024). Microglia from APOE ε4 carriers exhibit an exaggerated inflammatory response, including upregulation of cytokines and chemokines that can damage surrounding neurons and synapses (Shi et al., 2019; Yin et al., 2023; Rosenzweig et al., 2024). Furthermore, APOE ε4 impairs microglial capacity to clear Aβ and cellular debris, promoting plaque accumulation and contributing to a toxic environment that hastens neurodegeneration. Another key player is TREM2 (Triggering Receptor Expressed on Myeloid Cells 2), a receptor that regulates microglial activation in response to neuronal injury and amyloid deposition (Ulland and Colonna, 2018). TREM2 is critical for enabling microglia to transition into a “disease-associated” phenotype that facilitates the engulfment of Aβ plaques and cellular debris (Wang et al., 2022). However, loss-of-function variants in TREM2 significantly impair this response (Ulland and Colonna, 2018; Deczkowska et al., 2018; Deczkowska et al., 2020; Nugent et al., 2020). Individuals with such variants exhibit reduced microglial clustering around plaques and compromised containment of Aβ, which contributes to increased neuronal damage and disease progression (Ulland and Colonna, 2018; Deczkowska et al., 2018; Deczkowska et al., 2020; Nugent et al., 2020). CD33 is another important immune gene implicated in AD. It encodes a sialic acid-binding immunoglobulin-like lectin that functions as a negative regulator of microglial phagocytosis (Eskandari-Sedighi et al., 2024; Griciuc et al., 2013; Malik et al., 2013). Risk variants in CD33 are associated with increased gene expression in microglia, leading to a suppression of Aβ clearance (Eskandari-Sedighi et al., 2024; Griciuc et al., 2013; Malik et al., 2013). This anti-phagocytic effect creates a permissive environment for plaque accumulation and sustained inflammation, exacerbating neurodegenerative processes.

CLU, or clusterin, is a chaperone protein involved in lipid transport, apoptosis, and regulation of the complement cascade (Foster et al., 2019; Lish et al., 2025; Spatharas et al., 2022). In the brain, clusterin modulates glial responses and helps regulate complement activation, which is crucial for immune surveillance and synaptic pruning (Foster et al., 2019; Lish et al., 2025; Spatharas et al., 2022). Genetic variants in CLU are thought to disrupt these regulatory functions, promoting chronic glial activation and prolonged inflammation (Foster et al., 2019; Lish et al., 2025; Spatharas et al., 2022; Yu and Tan, 2012). Similarly, the CR1 gene encodes Complement Receptor 1, a key component in the classical complement pathway responsible for clearing immune complexes and cellular debris (Brouwers et al., 2012; Crehan et al., 2012; Kucukkilic et al., 2018). CR1 is also involved in mediating synaptic pruning via microglia. AD-associated variants in CR1 are believed to enhance complement activity, resulting in excessive and inappropriate elimination of synapses and heightened neuroinflammatory signaling (Brouwers et al., 2012; Crehan et al., 2012; Kucukkilic et al., 2018; Daskoulidou et al., 2023). This aberrant synaptic loss may contribute directly to the cognitive decline observed in AD. Finally, INPP5D, which encodes the phosphatase SHIP1, plays a critical role in negatively regulating immune signaling in microglia (Chou et al., 2023; Iguchi et al., 2023; Samuels et al., 2023; Tsai et al., 2021). SHIP1 acts downstream of several receptors, including TREM2, to limit overactivation of inflammatory pathways. Genetic variants in INPP5D associated with AD are thought to impair SHIP1 function, leading to heightened microglial activation and reduced capacity to resolve inflammation (Chou et al., 2023; Iguchi et al., 2023; Samuels et al., 2023; Tsai et al., 2021). This maladaptive immune environment may enhance the vulnerability of synapses and neurons to tau pathology and degeneration.

Collectively, these inflammation-related genetic risk factors contribute to a state of sustained microglial activation and impaired resolution of immune responses. This chronic inflammation disrupts normal homeostasis in the brain, promotes synaptic loss, and enhances tau pathology and neurodegeneration. As such, these genes represent both important biomarkers of AD risk and promising targets for therapeutic intervention aimed at modulating neuroimmune function.

#### Conserved immune-related mechanisms in aging humans and non-human primates

Immune activation during aging exhibits remarkably conserved features in both humans and NHPs. In humans, advanced age is associated with a shift toward a primed microglial phenotype, characterized by elevated expression of pro-inflammatory genes such as IL-1β, TNF-*α*, and MHC-II (Liddelow et al., 2017; Zhang et al., 2023). This phenotype is particularly evident in association cortices that are vulnerable in AD. NHPs, including aged rhesus macaques and marmosets, display similar microglial priming and regional vulnerability—especially in the prefrontal and entorhinal cortices—mirroring the inflammatory profile observed in humans (Beckman et al., 2021; Beckman et al., 2024; Rodriguez-Callejas et al., 2016; Sharma et al., 2019).

The role of TREM2 in mediating microglial response to amyloid is another conserved mechanism. In humans, loss-of-function variants in TREM2 weaken microglial plaque-associated clustering and amyloid containment (Ulland and Colonna, 2018; Deczkowska et al., 2018; Deczkowska et al., 2020; Nugent et al., 2020). Conversely, functional TREM2 supports microglial encapsulation of amyloid plaques, potentially limiting neuronal damage (Ulland and Colonna, 2018; Deczkowska et al., 2018; Deczkowska et al., 2020; Nugent et al., 2020; Parhizkar et al., 2019). Similarly, in aged macaques displaying early amyloid or tau pathology, TREM2 expression is upregulated around plaques, indicating preserved microglial responses across species (Beckman et al., 2024). The complement system cascade follows a parallel pattern. In aging humans, complement proteins such as C1q and C3 accumulate in synapse-rich regions, contributing to synaptic pruning and degeneration (Stephan et al., 2012; Stephan et al., 2013). NHPs also show age-related increases in these proteins in vulnerable cortical areas like the dlPFC, revealing a similar complement-driven mechanism of synaptic decline (Datta et al., 2020). Pro-inflammatory cytokines likewise mark both human and NHP aging. Increased levels of IL-6, IL-1β, and TNF-α are well documented in human cerebrospinal fluid and cortical tissue in aging and AD (Brosseron et al., 2014; Swardfager et al., 2010). These elevations are mirrored by similar cytokine increases in aged macaque brains and CSF, further underlining the translational relevance of NHP models for neuroinflammatory processes (Beckman et al., 2021; Beckman et al., 2019). Enhanced expression of MHC-II and interferon signaling are also observed. In humans, aging and AD are characterized by upregulation of MHC-II on microglia, which suggests increased antigen presentation and immune activation (Gao et al., 2023; Bossers et al., 2010; Perlmutter et al., 1992; Valiukas et al., 2025). Aged monkeys show comparable increases in MHC-II-positive microglia within vulnerable cortical regions (Sheffield and Berman, 1998). Furthermore, peripheral immune cell infiltration is another inflammatory signature that is preserved across species. Human studies report modest increases in T-cell infiltration into aged and AD brains (Gate et al., 2020; Jorfi et al., 2023; Zeng et al., 2024). NHPs exhibit a comparable but generally lower-level infiltration of CD3^+^ T cells in aging white matter, often associated with microglial reactivity and cognitive decline (Batterman et al., 2021).

Emerging evidence suggests sex-specific differences in immune aging (DeCasien et al., 2024; Sabogal-Guaqueta et al., 2023). In humans, females demonstrate stronger age-related immune activation in the brain, potentially contributing to their elevated AD risk. Likewise, sex-based differences in microglial gene expression and reactivity are evident in aged monkeys, further supporting the relevance of NHP models to investigate these biological variations across both species (Edler et al., 2021; DeCasien et al., 2024; Sabogal-Guaqueta et al., 2023).

#### Key aspects of calcium and immune dysregulation based on research in aging rhesus macaques

Research in aging rhesus macaques has revealed key mechanistic insights into calcium dysregulation and immune-related signaling disturbances that are highly relevant to the pathogenesis of sAD (Datta and Arnsten, 2025). One major finding is that calcium homeostasis is disrupted in vulnerable cortical areas such as the ERC and dlPFC. Specifically, calcium “leak” from the SER via hyperphosphorylated RyR2 channels in vulnerable aged macaque neurons exacerbates calcium dysregulation (Paspalas et al., 2018; Datta et al., 2021). These “leaky” RyR2 channels have been observed in the brains of patients with sAD (Lacampagne et al., 2017), and can induce greater calcium conductance from the SER into the cytosol (Marx et al., 2000; Bellinger et al., 2008). This calcium “leak” is closely associated with the accumulation of pTau on the SER, suggesting a direct pathological link between tau pathology and intracellular calcium dysregulation in higher-order association cortices. The aging brain also shows elevated cAMP-calcium signaling due to multiple converging mechanisms. Calbindin, a calcium-binding protein that buffers intracellular calcium, is significantly reduced in aged pyramidal neurons in macaque dlPFC (Datta et al., 2021). Loss of calbindin is a particularly important marker of neuronal vulnerability. In both aged macaques and humans, calbindin levels are significantly reduced in the dlPFC, and this loss correlates with the presence of tau pathology (Datta et al., 2021; Hof and Morrison, 1991). Calbindin loss has also been observed in conditions that increase AD risk, such as chronic stress and COVID-19, suggesting it may serve as a convergent pathway linking environmental insults to neurodegeneration (Guo et al., 1998; Erraji-Benchekroun et al., 2005; Li et al., 2017; Reiken et al., 2022). At the same time, enzymes that degrade cAMP, such as phosphodiesterase 4A and 4D (PDE4A/D), and receptors that suppress cAMP production, such as the metabotropic glutamate receptor mGluR3, are also diminished (Carlyle et al., 2014; Datta et al., 2021; Datta et al., 2020; Hernandez et al., 2018). Moreover, aging leads to activation of MAP kinase-activated protein kinase 2 (MK2), which disrupts the anchoring of PDE4 to DISC1 scaffolds, further amplifying cAMP signaling (MacKenzie et al., 2011). The result is excessive intracellular calcium signaling that renders neurons more vulnerable to damage and degeneration.

Inflammatory processes further exacerbate this vulnerability by disrupting protective neuromodulatory systems. In particular, under conditions of inflammation, microglia express the enzyme glutamate carboxypeptidase II (GCPII), which degrades N-acetylaspartylglutamate (NAAG), a peptide co-transmitter that selectively activates mGluR3 (Zhang et al., 2016; Arteaga Cabeza et al., 2021). Loss of NAAG signaling weakens mGluR3-mediated suppression of cAMP production, further amplifying calcium signaling cascades (see section below). Notably, GCPII activity in the macaque dlPFC highly correlates with accumulation of pT217Tau, the pathological tau species implicated in early AD (Bathla et al., 2023). As mGluR3 have an expanded role in primates compared to rodents, the primate model is especially important for studying inflammatory mechanisms relevant to human that are not adequately modeled in rodents.

## Evolutionary expansion of mGluR3-NAAG-GCPII signaling: novel roles of GCPII inhibition with 2-MPPA to reduce early-stage tau pathology

As described above, mGluR3 have a new, regulatory role in primate association cortex that is especially important to cognition and cognitive disorders. Genetic studies emphasize the importance of mGluR3 and GCPII signaling to human cognition. For example, a loss-of-function in *GRM3* encoding mGluR3 is a risk factor for schizophrenia (Arnsten and Wang, 2020), and a gain-of-function alteration in *FOLH1*, which leads to excessive levels of GCPII, is associated with impaired cognitive abilities in humans (Zink et al., 2020). Research in macaques helps to explain why this signaling pathway has such importance to human cognition.

In contrast to rodents where mGluR3 are primarily presynaptic and inhibit glutamate release (Woo et al., 2022), research in the rhesus monkey dlPFC has revealed that mGluR3 are postsynaptic on dendritic spines, and they play a key role in regulating cAMP drive on calcium-K^+^ channel signaling (Figure 2A), thus maintaining the strength of excitatory connections involved in working memory (Jin et al., 2018; Jin et al., 2017; Yang et al., 2022; Arnsten and Wang, 2020). This spatial configuration with mGluR3 immunolabeling in postsynaptic compartments in dendritic spines and shafts is also expressed in macaque ERC layer II microcircuits, that are especially vulnerable in sAD (Datta et al., 2023).

Within dendritic spines, mGluR3s are predominantly localized on the spine membrane in close proximity to the spine apparatus—a specialized extension of the SER that regulates intracellular calcium dynamics within dendritic spines (Jin et al., 2018; Jin et al., 2017; Datta et al., 2023). This spatial arrangement places mGluR3 in an ideal position to modulate local calcium signaling. As described above, elevated cAMP-PKA activity can stimulate calcium release from the spine apparatus, which in turn promotes further cAMP production, creating a feedforward loop of calcium–cAMP–PKA signaling (Figure 3). PKA signaling also enhances calcium influx through NMDA receptors and L-type voltage-gated calcium channels like Cav_1.2_, leading to cytosolic calcium accumulation (Datta et al., 2024; Wang et al., 2013) (Figure 3). The physiological contributions of mGluR3s have been demonstrated through iontophoretic application of NAAG (the endogenous mGluR3 agonist) or GCPII inhibitors directly onto dlPFC neurons in behaving monkeys (Yang et al., 2022; Arnsten and Wang, 2020). Both treatments significantly increased task-related neuronal firing by suppressing the cAMP–PKA–K^+^ channel pathway. A dose-dependent relationship was observed, where higher GCPII activity (and thus lower NAAG availability) led to reduced neuronal firing, underscoring the regulatory role of this signaling pathway (Yang et al., 2022; Arnsten and Wang, 2020). Overall, under normal physiological conditions NAAG–mGluR3 signaling serves a protective role by suppressing excessive cAMP and calcium signaling, thereby enhancing the connectivity of primate higher cortical circuits, fundamentally differing from the inhibitory presynaptic role of mGluR3 in rodents.

In the aging primate brain, the GCPII-NAAG-mGluR3 pathway becomes compromised due to the upregulation of GCPII, which degrades NAAG (Bathla et al., 2023; Arnsten and Wang, 2020). Pharmacological inhibition of GCPII has emerged as a promising strategy to restore mGluR3 function and suppress pathogenic inflammatory and calcium signaling. Acute administration of GCPII inhibitors in aged macaques has been shown to restore neuronal firing in the dlPFC and improve working memory performance, providing direct functional evidence for therapeutic benefit. Supporting these findings, rodent studies demonstrate that GCPII inhibition enhances spatial memory and object recognition (Datta et al., 2021; Olszewski et al., 2017), reinforcing its cognitive benefits across species. Notably, the orally bioavailable GCPII inhibitor 2-MPPA is particularly well suited for translational use due to its favorable side effect profile, making it viable for long-term preventive administration in at-risk individuals.

In aged rhesus macaques, increased GCPII activity has been strongly correlated with elevated levels of pT217Tau, a pathological marker associated with early AD, suggesting that inflammation-induced loss of mGluR3 signaling may directly promote tau pathology (Bathla et al., 2023). Chronic treatment with 2-MPPA in aging rhesus macaques has led to a significant reduction in both GCPII activity and pT217Tau levels in the dlPFC and ERC, two brain regions highly vulnerable to early AD pathology (Bathla et al., 2023). Additionally, decreases in pT217Tau were also observed in blood plasma, indicating the potential of this biomarker for tracking treatment response non-invasively even in non-human primates (Bathla et al., 2023). These findings highlight the therapeutic promise of targeting GCPII to preserve mGluR3 signaling, regulate intracellular calcium, and ultimately protect neural circuits from tau-mediated degeneration (Datta and Arnsten, 2025). Given the conserved biology between macaques and humans in this signaling pathway, aging rhesus macaques represent a powerful translational model for evaluating early-stage AD interventions (Datta and Arnsten, 2025). The accumulated data strongly supports further investigation of 2-MPPA and related GCPII inhibitors as viable preventative treatments for sAD, particularly in individuals with inflammation-related risk factors.

## Current landscape of therapies targeting neuro-immune interactions in sAD

The current therapeutic landscape targeting neuro-immune interactions in sAD reflects a growing recognition that inflammatory signaling, microglial state transitions, and neuronal calcium dysregulation are deeply interconnected drivers of disease progression. Many emerging therapeutic strategies—while often framed around immune modulation or protein clearance—intersect mechanistically with calcium- and cAMP-dependent pathways that regulate synaptic plasticity, tau phosphorylation, and neuronal survival. Thus, immune-targeted therapies may exert downstream benefits by indirectly restoring calcium homeostasis in vulnerable association cortices.

Several anti-inflammatory and metabolic agents currently under investigation act upstream of calcium dysregulation by suppressing cytokine-driven kinase signaling. For example, NE3107, a small molecule inhibitor of the NF-κB/ERK axis, reduces MAPK activation and pro-inflammatory cytokines including TNFα, IFNγ, IL-1α, and TGF-β, while also enhancing insulin signaling (Haroon et al., 2024; Reading et al., 2021). Because cytokine-activated kinases such as ERK and PKA can potentiate calcium release from intracellular stores and amplify cAMP–calcium feedforward signaling, dampening these pathways may indirectly stabilize calcium dynamics in pyramidal neurons. Similarly, semaglutide, a GLP-1 receptor agonist originally developed for metabolic disease, has been associated with reduced dementia risk in diabetic populations (Norgaard et al., 2022), although recent Phase 3 clinical trials have yielded negative results. GLP-1 signaling has been shown to modulate neuroinflammation, mitochondrial function, and calcium handling, suggesting that metabolic–immune therapies may converge on shared calcium-regulatory mechanisms.

Microglia-focused biologics further highlight the intersection between immune pathways and neuronal calcium vulnerability. AL002c, a monoclonal IgG1 antibody acting as a TREM2 agonist, promotes microglial state transitions associated with phagocytosis and plaque containment (Wang et al., 2020). In animal models carrying the R47H TREM2 variant, chronic AL002c treatment reduced filamentous amyloid plaques, neuritic dystrophy, and microglial inflammatory responses while improving behavioral outcomes (Wang et al., 2020). Importantly, microglial encapsulation of plaques—the so-called “microglial barrier”—limits the diffusion of inflammatory mediators, reactive oxygen species, and synaptotoxic factors that can destabilize neuronal calcium signaling (Condello et al., 2018). Thus, TREM2-based strategies may indirectly protect synapses and dendritic calcium nanodomains by shaping the inflammatory microenvironment. Related immune pathways, including the complement cascade, further link neuroinflammation to synaptic and calcium-dependent pathology. Complement proteins such as C1q and C3 are upregulated with aging and AD and contribute to aberrant synaptic pruning. Excessive complement activation can weaken synaptic integrity and increase neuronal calcium load, thereby sensitizing circuits to tau phosphorylation and degeneration. Although complement-targeting therapies are still largely preclinical in AD, these mechanisms align closely with models in which inflammatory weakening of synaptic and calcium-regulatory systems precedes overt neurodegeneration.

Peripheral immune modulation also intersects with central neuroimmune–calcium pathways. For example, daratumumab, an FDA-approved anti-CD38 antibody, modulates CD38(+) CD8(+) T cells. Single-cell immune profiling in AD patients has revealed an expansion of CD8(+) effector memory T cells that negatively correlates with cognitive performance (Gate et al., 2020). Because peripheral immune activation can influence central cytokine levels, oxidative stress, and microglial reactivity, targeting these pathways may further reduce inflammatory amplification of neuronal calcium signaling.

Finally, our work on GCPII inhibition provides a direct mechanistic bridge between immune activation and calcium dysregulation. Inflammatory upregulation of GCPII degrades NAAG and weakens mGluR3-mediated suppression of cAMP–calcium signaling in primate association cortex, thereby promoting tau pathology. Therapeutic strategies that restore neuromodulatory control of calcium signaling may therefore complement microglial- and cytokine-focused interventions. Collectively, these emerging therapies suggest that successful disease modification in sAD may require coordinated targeting of immune pathways and the calcium–cAMP signaling cascades through which inflammation exerts its most deleterious effects on vulnerable cortical circuits.

## Conclusion

Neuroinflammation-focused therapeutic strategies in AD are rapidly advancing, with efforts ranging from microglial modulation to cytokine regulation and synaptic protection. Although some clinical approaches have faced setbacks, the therapeutic pipeline continues to expand, underscoring the centrality of immune mechanisms in disease progression. Importantly, NHPs provide a critical bridge between rodent models and humans for the development of these therapies. Unlike rodents, NHPs naturally develop AD-like pathology, including amyloid and tau accumulation within the same association cortices affected in humans, and they exhibit age-related neuroinflammation that closely parallels human disease. Future work integrating human iPSC-based models across ApoE genotypes, as well as emerging humanized ApoE non-human primate approaches, will be important for mechanistic testing of neuro-immune hypotheses. Moreover, NHPs share highly conserved immune gene expression profiles and microglial responses, while supporting translational biomarker validation such as plasma and CSF pT217Tau. Their unique convergence of immune and neural features, combined with cognitive complexity, makes NHPs indispensable for testing neuroimmune-targeted interventions and predicting both efficacy and safety in humans.

## References

[ref1] Albuquerque E. X. Schwarcz R. (2013). Kynurenic acid as an antagonist of alpha7 nicotinic acetylcholine receptors in the brain: facts and challenges. Biochem. Pharmacol. 85, 1027–1032. doi: 10.1016/j.bcp.2012.12.014, 23270993 PMC3721521

[ref2] Almulla A. F. Supasitthumrong T. Amrapala A. Tunvirachaisakul C. Jaleel A. K. A. Oxenkrug G. . (2022). The tryptophan catabolite or kynurenine pathway in Alzheimer's disease: a systematic review and meta-analysis. J Alzheimer's Dis 88, 1325–1339. doi: 10.3233/JAD-220295, 35786655

[ref3] Area-Gomez E. Schon E. A. (2017). On the pathogenesis of Alzheimer's disease: the MAM hypothesis. FASEB J. 31, 864–867. doi: 10.1096/fj.201601309, 28246299 PMC6191063

[ref4] Arige V. Yule D. I. (2022). Spatial and temporal crosstalk between the cAMP and ca(2+) signaling systems. Biochim. Biophys. Acta, Mol. Cell Res. 1869:119293. doi: 10.1016/j.bbamcr.2022.119293, 35588944

[ref5] Arion D. Enwright J. F. Gonzalez-Burgos G. Lewis D. A. (2023). Differential gene expression between callosal and ipsilateral projection neurons in the monkey dorsolateral prefrontal and posterior parietal cortices. Cereb. Cortex 33, 1581–1594. doi: 10.1093/cercor/bhac157, 35441221 PMC9977376

[ref6] Arnsten A. F. T. Datta D. (2024). Characterizing the Most vulnerable prefrontal cortical neurons in schizophrenia. Am. J. Psychiatry 181, 861–864. doi: 10.1176/appi.ajp.20240731, 39350618 PMC11714303

[ref7] Arnsten A. F. T. Datta D. Del Tredici K. Braak H. (2021). Hypothesis: tau pathology is an initiating factor in sporadic Alzheimer’s disease. Alzheimers Dement. 17, 115–124. doi: 10.1002/alz.12192, 33075193 PMC7983919

[ref8] Arnsten A. F. T. Datta D. Leslie S. Yang S. T. Wang M. Nairn A. C. (2019). Alzheimer's-like pathology in aging rhesus macaques: unique opportunity to study the etiology and treatment of Alzheimer's disease. Proc. Natl. Acad. Sci. USA 116, 26230–26238. doi: 10.1073/pnas.1903671116, 31871209 PMC6936707

[ref9] Arnsten A. F. T. Datta D. Preuss T. M. (2021). Studies of aging nonhuman primates illuminate the etiology of early-stage Alzheimer's-like neuropathology: an evolutionary perspective. Am. J. Primatol. 83:e23254. doi: 10.1002/ajp.23254, 33960505 PMC8550995

[ref10] Arnsten A. F. T. Datta D. Wang M. (2021). The genie in the bottle-magnified calcium signaling in dorsolateral prefrontal cortex. Mol. Psychiatry 26, 3684–3700. doi: 10.1038/s41380-020-00973-3, 33319854 PMC8203737

[ref11] Arnsten A. F. T. Del Tredici K. Barthelemy N. R. Gabitto M. van Dyck C. H. Lein E. . (2025). An integrated view of the relationships between amyloid, tau, and inflammatory pathophysiology in Alzheimer's disease. Alzheimers Dement. 21:e70404. doi: 10.1002/alz.70404, 40767321 PMC12326325

[ref12] Arnsten A. F. T. Wang M. (2020). The evolutionary expansion of mGluR3-NAAG-GCPII signaling: relevance to human intelligence and cognitive disorders. Am. J. Psychiatry 177, 1103–1106. doi: 10.1176/appi.ajp.2020.20101458, 33256450

[ref13] Arnsten A. F. Wang M. J. Paspalas C. D. (2012). Neuromodulation of thought: flexibilities and vulnerabilities in prefrontal cortical network synapses. Neuron 76, 223–239. doi: 10.1016/j.neuron.2012.08.038, 23040817 PMC3488343

[ref14] Arnsten A. F. T. Woo E. Yang S. Wang M. Datta D. (2022). Unusual molecular regulation of dorsolateral prefrontal cortex layer III synapses increases vulnerability to genetic and environmental insults in schizophrenia. Biol. Psychiatry 92, 480–490. doi: 10.1016/j.biopsych.2022.02.003, 35305820 PMC9372235

[ref15] Arteaga Cabeza O. Zhang Z. Smith Khoury E. Sheldon R. A. Sharma A. Zhang F. . (2021). Neuroprotective effects of a dendrimer-based glutamate carboxypeptidase inhibitor on superoxide dismutase transgenic mice after neonatal hypoxic-ischemic brain injury. Neurobiol. Dis. 148:105201. doi: 10.1016/j.nbd.2020.105201, 33271328 PMC8351403

[ref16] Badawy A. A. (2017). Kynurenine pathway of tryptophan metabolism: regulatory and functional aspects. Int J Tryptophan Res 10:1178646917691938. doi: 10.1177/1178646917691938, 28469468 PMC5398323

[ref17] Badawy A. A. (2023). The kynurenine pathway of tryptophan metabolism: a neglected therapeutic target of COVID-19 pathophysiology and immunotherapy. Biosci. Rep. 43. doi: 10.1042/BSR20230595, 37486805 PMC10407158

[ref18] Barthelemy N. R. Li Y. Joseph-Mathurin N. Gordon B. A. Hassenstab J. Benzinger T. L. S. . (2020). A soluble phosphorylated tau signature links tau, amyloid and the evolution of stages of dominantly inherited Alzheimer's disease. Nat. Med. 26, 398–407. doi: 10.1038/s41591-020-0781-z, 32161412 PMC7309367

[ref19] Barthélemy N. R. Salvadó G. Schindler S. He Y. Janelidze S. Collij L. E. . (2024). Highly accurate blood test for Alzheimer's disease comparable or superior to clinical CSF tests. Nat. Med. 30, 1085–1095. doi: 10.1038/s41591-024-02869-z, 38382645 PMC11031399

[ref20] Bathla S. Datta D. Liang F. Barthelemy N. Wiseman R. Slusher B. S. . (2023). Chronic GCPII (glutamate-carboxypeptidase-II) inhibition reduces pT217Tau levels in the entorhinal and dorsolateral prefrontal cortices of aged macaques. Alzheimers Dement (N Y) 9:e12431. doi: 10.1002/trc2.12431, 37915375 PMC10617575

[ref21] Batterman K. V. Cabrera P. E. Moore T. L. Rosene D. L. (2021). T cells actively infiltrate the white matter of the aging monkey brain in relation to increased microglial reactivity and cognitive decline. Front. Immunol. 12:607691. doi: 10.3389/fimmu.2021.607691, 33664743 PMC7920950

[ref22] Beall M. J. Lewis D. A. (1992). Heterogeneity of layer II neurons in human entorhinal cortex. J. Comp. Neurol. 321, 241–266. doi: 10.1002/cne.903210206, 1500542

[ref23] Beckman D. Chakrabarty P. Ott S. Dao A. Zhou E. Janssen W. G. . (2021). A novel tau-based rhesus monkey model of Alzheimer's pathogenesis. Alzheimers Dement. 17, 933–945. doi: 10.1002/alz.12318, 33734581 PMC8252011

[ref24] Beckman D. Diniz G. B. Ott S. Hobson B. Chaudhari A. J. Muller S. . (2024). Temporal progression of tau pathology and neuroinflammation in a rhesus monkey model of Alzheimer's disease. Alzheimers Dement. 20, 5198–5219. doi: 10.1002/alz.13868, 39030748 PMC11350056

[ref25] Beckman D. Ott S. Donis-Cox K. Janssen W. G. Bliss-Moreau E. Rudebeck P. H. . (2019). Oligomeric Abeta in the monkey brain impacts synaptic integrity and induces accelerated cortical aging. Proc. Natl. Acad. Sci. USA 116, 26239–26246. doi: 10.1073/pnas.1902301116, 31871145 PMC6936351

[ref26] Bekkers J. M. Stevens C. F. (1989). NMDA and non-NMDA receptors are co-localized at individual excitatory synapses in cultured rat hippocampus. Nature 341, 230–233. doi: 10.1038/341230a0, 2571090

[ref27] Bellinger A. M. Reiken S. Dura M. Murphy P. W. Deng S. X. Landry D. W. . (2008). Remodeling of ryanodine receptor complex causes "leaky" channels: a molecular mechanism for decreased exercise capacity. Proc. Natl. Acad. Sci. USA 105, 2198–2202. doi: 10.1073/pnas.0711074105, 18268335 PMC2538898

[ref28] Bettcher B. M. Tansey M. G. Dorothee G. Heneka M. T. (2021). Peripheral and central immune system crosstalk in Alzheimer disease - a research prospectus. Nat. Rev. Neurol. 17, 689–701. doi: 10.1038/s41582-021-00549-x, 34522039 PMC8439173

[ref29] Bian Y. Kawabata R. Enwright J. F. Tsubomoto M. Okuda T. Kamikawa K. . (2024). Expression of activity-regulated transcripts in pyramidal neurons across the cortical visuospatial working memory network in unaffected comparison individuals and individuals with schizophrenia. Psychiatry Res. 339:116084. doi: 10.1016/j.psychres.2024.116084, 39033685

[ref30] Bossers K. Wirz K. T. Meerhoff G. F. Essing A. H. van Dongen J. W. Houba P. . (2010). Concerted changes in transcripts in the prefrontal cortex precede neuropathology in Alzheimer's disease. Brain 133, 3699–3723. doi: 10.1093/brain/awq258, 20889584

[ref31] Braak H. Braak E. (1991). Neuropathological stageing of Alzheimer-related changes. Acta Neuropathol. 82, 239–259. doi: 10.1007/bf00308809, 1759558

[ref32] Braak H. Del Trecidi K. (2015). Neuroanatomy and pathology of sporadic Alzheimer's disease. Adv. Anat. Embryol. Cell Biol. 215, 1–162, 25920101

[ref33] Braak H. Del Tredici K. (2011). Alzheimer's pathogenesis: is there neuron-to-neuron propagation? Acta Neuropathol. 121, 589–595. doi: 10.1007/s00401-011-0825-z, 21516512

[ref34] Braak H. Del Tredici K. (2015). The preclinical phase of the pathological process underlying sporadic Alzheimer's disease. Brain 138, 2814–2833. doi: 10.1093/brain/awv236, 26283673

[ref35] Braak H. Thal D. R. Ghebremedhin E. Del Tredici K. (2011). Stages of the pathologic process in Alzheimer disease: age categories from 1 to 100 years. J. Neuropathol. Exp. Neurol. 70, 960–969. doi: 10.1097/NEN.0b013e318232a379, 22002422

[ref36] Brosseron F. Krauthausen M. Kummer M. Heneka M. T. (2014). Body fluid cytokine levels in mild cognitive impairment and Alzheimer's disease: a comparative overview. Mol. Neurobiol. 50, 534–544. doi: 10.1007/s12035-014-8657-1, 24567119 PMC4182618

[ref37] Brouwers N. Van Cauwenberghe C. Engelborghs S. Lambert J. C. Bettens K. Le Bastard N. . (2012). Alzheimer risk associated with a copy number variation in the complement receptor 1 increasing C3b/C4b binding sites. Mol. Psychiatry 17, 223–233. doi: 10.1038/mp.2011.24, 21403675 PMC3265835

[ref38] Bruno A. M. Huang J. Y. Bennett D. A. Marr R. A. Hastings M. L. Stutzmann G. E. (2012). Altered ryanodine receptor expression in mild cognitive impairment and Alzheimer's disease. Neurobiol. Aging 33, 1001 e1–1001 e6. doi: 10.1016/j.neurobiolaging.2011.03.011, 21531043 PMC3160507

[ref39] Burt J. B. Demirtaş M. Eckner W. J. Navejar N. M. Ji J. L. Martin W. J. . (2018). Hierarchy of transcriptomic specialization across human cortex captured by structural neuroimaging topography. Nature Neurosci 21, 1251–1259. doi: 10.1038/s41593-018-0195-0, 30082915 PMC6119093

[ref40] Carlyle B. C. Kitchen R. R. Kanyo J. E. Voss E. Z. Pletikos M. Sousa A. M. M. . (2017). A multiregional proteomic survey of the postnatal human brain. Nat. Neurosci. 20, 1787–1795. doi: 10.1038/s41593-017-0011-2, 29184206 PMC5894337

[ref41] Carlyle B. C. Nairn A. C. Wang M. Yang Y. Jin L. E. Simen A. A. . (2014). cAMP-PKA phosphorylation of tau confers risk for degeneration in aging association cortex. Proc. Natl. Acad. Sci. USA 111, 5036–5041. doi: 10.1073/pnas.1322360111, 24707050 PMC3977284

[ref42] Chakroborty S. Goussakov I. Miller M. B. Stutzmann G. E. (2009). Deviant ryanodine receptor-mediated calcium release resets synaptic homeostasis in presymptomatic 3xTg-AD mice. J. Neurosci. 29, 9458–9470. doi: 10.1523/JNEUROSCI.2047-09.2009, 19641109 PMC6666542

[ref43] Chatterjee P. Zetterberg H. Goozee K. Lim C. K. Jacobs K. R. Ashton N. J. . (2019). Plasma neurofilament light chain and amyloid-β are associated with the kynurenine pathway metabolites in preclinical Alzheimer’s disease. J. Neuroinflammation 16:186. doi: 10.1186/s12974-019-1567-4, 31601232 PMC6788092

[ref44] Cheung K. H. Mei L. Mak D. O. Hayashi I. Iwatsubo T. Kang D. E. . (2010). Gain-of-function enhancement of IP3 receptor modal gating by familial Alzheimer's disease-linked presenilin mutants in human cells and mouse neurons. Sci. Signal. 3:ra22. doi: 10.1126/scisignal.2000818, 20332427 PMC2898196

[ref45] Chiou K. L. DeCasien A. R. Rees K. P. Testard C. Spurrell C. H. Gogate A. A. . (2022). Multiregion transcriptomic profiling of the primate brain reveals signatures of aging and the social environment. Nat. Neurosci. 25, 1714–1723. doi: 10.1038/s41593-022-01197-0, 36424430 PMC10055353

[ref46] Chou V. Pearse R. V. 2nd Aylward A. J. Ashour N. Taga M. Terzioglu G. . (2023). INPP5D regulates inflammasome activation in human microglia. Nat. Commun. 14:7552. doi: 10.1038/s41467-023-42819-w, 38016942 PMC10684891

[ref47] Condello C. Yuan P. Grutzendler J. (2018). Microglia-mediated neuroprotection, TREM2, and Alzheimer's disease: evidence from optical imaging. Biol. Psychiatry 83, 377–387. doi: 10.1016/j.biopsych.2017.10.007, 29169609 PMC5767550

[ref48] Congdon E. E. Ji C. Tetlow A. M. Jiang Y. Sigurdsson E. M. (2023). Tau-targeting therapies for Alzheimer disease: current status and future directions. Nat. Rev. Neurol. 19, 715–736. doi: 10.1038/s41582-023-00883-2, 37875627 PMC10965012

[ref49] Cooper L. L. Li W. Lu Y. Centracchio J. Terentyeva R. Koren G. . (2013). Redox modification of ryanodine receptors by mitochondria-derived reactive oxygen species contributes to aberrant Ca2+ handling in ageing rabbit hearts. J. Physiol. 591, 5895–5911. doi: 10.1113/jphysiol.2013.260521, 24042501 PMC3872760

[ref50] Corder E. H. Saunders A. M. Risch N. J. Strittmatter W. J. Schmechel D. E. Gaskell P. C. Jr. . (1994). Protective effect of apolipoprotein E type 2 allele for late onset Alzheimer disease. Nat. Genet. 7, 180–184. doi: 10.1038/ng0694-180, 7920638

[ref51] Crehan H. Holton P. Wray S. Pocock J. Guerreiro R. Hardy J. (2012). Complement receptor 1 (CR1) and Alzheimer's disease. Immunobiology 217, 244–250. doi: 10.1016/j.imbio.2011.07.017, 21840620

[ref52] Cysique L. A. Jakabek D. Bracken S. G. Allen-Davidian Y. Heng B. Chow S. . (2023). The kynurenine pathway relates to post-acute COVID-19 objective cognitive impairment and PASC. Ann. Clin. Transl. Neurol. 10, 1338–1352. doi: 10.1002/acn3.51825, 37318955 PMC10424655

[ref53] Daskoulidou N. Shaw B. Torvell M. Watkins L. Cope E. L. Carpanini S. M. . (2023). Complement receptor 1 is expressed on brain cells and in the human brain. Glia 71, 1522–1535. doi: 10.1002/glia.24355, 36825534 PMC10953339

[ref54] Datta D. Arnsten A. F. T. (2018). Unique molecular regulation of higher-order prefrontal cortical circuits: insights into the neurobiology of schizophrenia. ACS Chem. Neurosci. 9, 2127–2145. doi: 10.1021/acschemneuro.7b00505, 29470055 PMC6119516

[ref55] Datta D. Arnsten A. F. T. (2019). Loss of prefrontal cortical higher cognition with uncontrollable stress: molecular mechanisms, changes with age, and relevance to treatment. Brain Sci. 9. doi: 10.3390/brainsci9050113, 31108855 PMC6562841

[ref56] Datta D. Arnsten A. F. T. (2025). The etiology and prevention of early-stage tau pathology in higher cortical circuits: insights from aging rhesus macaques. Alzheimers Dement. 21:e14477. doi: 10.1002/alz.14477, 39776253 PMC11848412

[ref57] Datta D. Enwright J. F. Arion D. Paspalas C. D. Morozov Y. M. Lewis D. A. . (2020). Mapping phosphodiesterase 4D (PDE4D) in macaque dorsolateral prefrontal cortex: postsynaptic compartmentalization in layer III pyramidal cell circuits. Front. Neuroanat. 14:578483. doi: 10.3389/fnana.2020.578483, 33328902 PMC7714912

[ref58] Datta D. Leslie S. N. Morozov Y. M. Duque A. Rakic P. van Dyck C. H. . (2020). Classical complement cascade initiating C1q protein within neurons in the aged rhesus macaque dorsolateral prefrontal cortex. J. Neuroinflammation 17:8. doi: 10.1186/s12974-019-1683-1, 31906973 PMC6945481

[ref59] Datta D. Leslie S. N. Wang M. Morozov Y. M. Yang S. Mentone S. . (2021). Age-related calcium dysregulation linked with tau pathology and impaired cognition in non-human primates. Alzheimers Dement. 17, 920–932. doi: 10.1002/alz.12325, 33829643 PMC8195842

[ref60] Datta D. Leslie S. N. Woo E. Amancharla N. Elmansy A. Lepe M. . (2021). Glutamate carboxypeptidase II in aging rat prefrontal cortex impairs working memory performance. Front. Aging Neurosci. 13:760270. doi: 10.3389/fnagi.2021.760270, 34867287 PMC8634091

[ref61] Datta D. Perone I. Morozov Y. M. Arellano J. Duque A. Rakic P. . (2023). Localization of PDE4D, HCN1 channels, and mGluR3 in rhesus macaque entorhinal cortex may confer vulnerability in Alzheimer's disease. Cereb. Cortex 33, 11501–11516. doi: 10.1093/cercor/bhad382, 37874022 PMC10724870

[ref62] Datta D. Perone I. Wijegunawardana D. Liang F. Morozov Y. M. Arellano J. . (2024). Nanoscale imaging of pT217-tau in aged rhesus macaque entorhinal and dorsolateral prefrontal cortex: evidence of interneuronal trafficking and early-stage neurodegeneration. Alzheimers Dement. 20, 2843–2860. doi: 10.1002/alz.13737, 38445818 PMC11032534

[ref63] Datta D. Yang S. T. Galvin V. C. Solder J. Luo F. Morozov Y. M. . (2019). Noradrenergic alpha1-Adrenoceptor actions in the primate dorsolateral prefrontal cortex. J. Neurosci. 39, 2722–2734. doi: 10.1523/JNEUROSCI.2472-18.2019, 30755491 PMC6445993

[ref64] Datta D. Yang S. Joyce M. K. P. Woo E. McCarroll S. A. Gonzalez-Burgos G. . (2024). Key roles of CACNA1C/Cav1.2 and CALB1/Calbindin in prefrontal neurons altered in cognitive disorders. JAMA Psychiatr. doi: 10.1001/jamapsychiatry.2024.1112 PMC11112502 38776078

[ref65] DeCasien A. R. Chiou K. L. Testard C. Mercer A. Negron-Del Valle J. E. Bauman Surratt S. E. . (2024). Evolutionary and biomedical implications of sex differences in the primate brain transcriptome. Cell Genom. 4:100589. doi: 10.1016/j.xgen.2024.100589 38942023 PMC11293591

[ref66] Deczkowska A. Keren-Shaul H. Weiner A. Colonna M. Schwartz M. Amit I. (2018). Disease-associated microglia: a universal immune sensor of neurodegeneration. Cell 173, 1073–1081. doi: 10.1016/j.cell.2018.05.003, 29775591

[ref67] Deczkowska A. Weiner A. Amit I. (2020). The physiology, pathology, and potential therapeutic applications of the TREM2 signaling pathway. Cell 181, 1207–1217. doi: 10.1016/j.cell.2020.05.003, 32531244

[ref68] Defelipe J. (2011). The evolution of the brain, the human nature of cortical circuits, and intellectual creativity. Front. Neuroanat. 5:29. doi: 10.3389/fnana.2011.00029, 21647212 PMC3098448

[ref69] DeKosky S. T. Scheff S. W. (1990). Synapse loss in frontal cortex biopsies in Alzheimer's disease: correlation with cognitive severity. Ann. Neurol. 27, 457–464. doi: 10.1002/ana.410270502, 2360787

[ref70] Dreses-Werringloer U. Lambert J. C. Vingtdeux V. Zhao H. Vais H. Siebert A. . (2008). A polymorphism in CALHM1 influences Ca2+ homeostasis, Abeta levels, and Alzheimer's disease risk. Cell 133, 1149–1161. doi: 10.1016/j.cell.2008.05.048, 18585350 PMC2577842

[ref71] Edler M. K. Mhatre-Winters I. Richardson J. R. (2021). Microglia in aging and Alzheimer's disease: a comparative species review. Cells 10. doi: 10.3390/cells10051138, 34066847 PMC8150617

[ref72] Ekinci F. J. Malik K. U. Shea T. B. (1999). Activation of the L voltage-sensitive calcium channel by mitogen-activated protein (MAP) kinase following exposure of neuronal cells to beta-amyloid. MAP kinase mediates beta-amyloid-induced neurodegeneration. J. Biol. Chem. 274, 30322–30327. doi: 10.1074/jbc.274.42.30322, 10514528

[ref73] Elston G. N. (2000). Pyramidal cells of the frontal lobe: all the more spinous to think with. J. Neurosci. 20:RC95. doi: 10.1523/jneurosci.20-18-j0002.2000, 10974092 PMC6772841

[ref74] Elston G. N. (2003). Cortex, cognition and the cell: new insights into the pyramidal neuron and prefrontal function. Cereb. Cortex 13, 1124–1138. doi: 10.1093/cercor/bhg093, 14576205

[ref75] Elston G. N. Benavides-Piccione R. DeFelipe J. (2001). The pyramidal cell in cognition: a comparative study in human and monkey. J. Neurosci. 21:RC163. doi: 10.1523/JNEUROSCI.21-17-j0002.2001, 11511694 PMC6763111

[ref76] Elston G. N. Benavides-Piccione R. Elston A. Manger P. R. Defelipe J. (2011). Pyramidal cells in prefrontal cortex of primates: marked differences in neuronal structure among species. Front. Neuroanat. 5:2. doi: 10.3389/fnana.2011.00002 21347276 PMC3039119

[ref77] Elston G. N. Benavides-Piccione R. Elston A. Zietsch B. Defelipe J. Manger P. . (2006). Specializations of the granular prefrontal cortex of primates: implications for cognitive processing. Anat Rec A Discov Mol Cell Evol Biol 288, 26–35. doi: 10.1002/ar.a.20278, 16342214

[ref78] Enwright J. F. III Arion D. MacDonald W. A. Elbakri R. Pan Y. Vyas G. . (2022). Differential gene expression in layer 3 pyramidal neurons across 3 regions of the human cortical visual spatial working memory network. Cereb. Cortex 32, 5216–5229. doi: 10.1093/cercor/bhac009, 35106549 PMC9667185

[ref79] Erraji-Benchekroun L. Underwood M. D. Arango V. Galfalvy H. Pavlidis P. Smyrniotopoulos P. . (2005). Molecular aging in human prefrontal cortex is selective and continuous throughout adult life. Biol. Psychiatry 57, 549–558. doi: 10.1016/j.biopsych.2004.10.034, 15737671

[ref80] Eskandari-Sedighi G. Crichton M. Zia S. Gomez-Cardona E. Cortez L. M. Patel Z. H. . (2024). Alzheimer's disease associated isoforms of human CD33 distinctively modulate microglial cell responses in 5XFAD mice. Mol. Neurodegener. 19:42. doi: 10.1186/s13024-024-00734-8, 38802940 PMC11129479

[ref81] Fitzpatrick A. W. P. Falcon B. He S. Murzin A. G. Murshudov G. Garringer H. J. . (2017). Cryo-EM structures of tau filaments from Alzheimer's disease. Nature 547, 185–190. doi: 10.1038/nature23002, 28678775 PMC5552202

[ref82] Foster E. M. Dangla-Valls A. Lovestone S. Ribe E. M. Buckley N. J. (2019). Clusterin in Alzheimer's disease: mechanisms, genetics, and lessons from other pathologies. Front. Neurosci. 13:164. doi: 10.3389/fnins.2019.00164, 30872998 PMC6403191

[ref83] Gao C. Jiang J. Tan Y. Chen S. (2023). Microglia in neurodegenerative diseases: mechanism and potential therapeutic targets. Signal Transduct. Target. Ther. 8:359. doi: 10.1038/s41392-023-01588-0, 37735487 PMC10514343

[ref84] Gate D. Saligrama N. Leventhal O. Yang A. C. Unger M. S. Middeldorp J. . (2020). Clonally expanded CD8 T cells patrol the cerebrospinal fluid in Alzheimer's disease. Nature 577, 399–404. doi: 10.1038/s41586-019-1895-7, 31915375 PMC7445078

[ref85] Geirsdottir L. David E. Keren-Shaul H. Weiner A. Bohlen S. C. Neuber J. . (2019). Cross-species single-cell analysis reveals divergence of the primate microglia program. Cell 179, 1609–1622 e16. doi: 10.1016/j.cell.2019.11.010, 31835035

[ref86] Giannakopoulos P. Herrmann F. R. Bussière T. Bouras C. Kövari E. Perl D. P. . (2003). Tangle and neuron numbers, but not amyloid load, predict cognitive status in Alzheimer's disease. Neurology 60, 1495–1500. doi: 10.1212/01.wnl.0000063311.58879.01, 12743238

[ref87] Gibbons G. S. Lee V. M. Y. Trojanowski J. Q. (2019). Mechanisms of cell-to-cell transmission of pathological tau: a review. JAMA Neurol. 76, 101–108. doi: 10.1001/jamaneurol.2018.2505, 30193298 PMC6382549

[ref88] Gibson G. E. Thakkar A. (2017). Interactions of mitochondria/metabolism and calcium regulation in Alzheimer's disease: a Calcinist point of view. Neurochem. Res. 42, 1636–1648. doi: 10.1007/s11064-017-2182-3, 28181072 PMC5451308

[ref89] Gilman J. P. Medalla M. Luebke J. I. (2017). Area-specific features of pyramidal neurons-a comparative study in mouse and Rhesus monkey. Cereb. Cortex 27, 2078–2094. doi: 10.1093/cercor/bhw062, 26965903 PMC6059164

[ref90] Giorgi C. Marchi S. Simoes I. C. M. Ren Z. Morciano G. Perrone M. . (2018). Mitochondria and reactive oxygen species in aging and age-related diseases. Int. Rev. Cell Mol. Biol. 340, 209–344. doi: 10.1016/bs.ircmb.2018.05.006, 30072092 PMC8127332

[ref91] Glantz L. A. Lewis D. A. (2000). Decreased dendritic spine density on prefrontal cortical pyramidal neurons in schizophrenia. Arch. Gen. Psychiatry 57, 65–73. doi: 10.1001/archpsyc.57.1.65, 10632234

[ref92] Gonneaud J. Arenaza-Urquijo E. M. Fouquet M. Perrotin A. Fradin S. de La Sayette V. . (2016). Relative effect of APOE ε4 on neuroimaging biomarker changes across the lifespan. Neurology 87, 1696–1703. doi: 10.1212/wnl.0000000000003234, 27683850

[ref93] Goussakov I. Miller M. B. Stutzmann G. E. (2010). NMDA-mediated ca(2+) influx drives aberrant ryanodine receptor activation in dendrites of young Alzheimer's disease mice. J. Neurosci. 30, 12128–12137. doi: 10.1523/JNEUROSCI.2474-10.2010, 20826675 PMC2944253

[ref94] Griciuc A. Serrano-Pozo A. Parrado A. R. Lesinski A. N. Asselin C. N. Mullin K. . (2013). Alzheimer's disease risk gene CD33 inhibits microglial uptake of amyloid beta. Neuron 78, 631–643. doi: 10.1016/j.neuron.2013.04.014, 23623698 PMC3706457

[ref95] Grynspan F. Griffin W. R. Cataldo A. Katayama S. Nixon R. A. (1997). Active site-directed antibodies identify calpain II as an early-appearing and pervasive component of neurofibrillary pathology in Alzheimer's disease. Brain Res. 763, 145–158. doi: 10.1016/s0006-8993(97)00384-3, 9296555

[ref96] Guo Q. Christakos S. Robinson N. Mattson M. P. (1998). Calbindin D28k blocks the proapoptotic actions of mutant presenilin 1: reduced oxidative stress and preserved mitochondrial function. Proc. Natl. Acad. Sci. USA 95, 3227–3232. doi: 10.1073/pnas.95.6.3227, 9501245 PMC19724

[ref97] Haage V. De Jager P. L. (2022). Neuroimmune contributions to Alzheimer's disease: a focus on human data. Mol. Psychiatry 27, 3164–3181. doi: 10.1038/s41380-022-01637-0, 35668160 PMC9168642

[ref98] Hardingham G. E. Pruunsild P. Greenberg M. E. Bading H. (2018). Lineage divergence of activity-driven transcription and evolution of cognitive ability. Nat. Rev. Neurosci. 19, 9–15. doi: 10.1038/nrn.2017.138, 29167525

[ref99] Haroon J. Jordan K. Mahdavi K. Rindner E. Becerra S. Surya J. R. . (2024). A phase 2, open-label study of anti-inflammatory NE3107 in patients with dementias. Medicine (Baltimore) 103:e39027. doi: 10.1097/MD.0000000000039027, 39058809 PMC11272329

[ref100] Hernandez C. M. McQuail J. A. Schwabe M. R. Burke S. N. Setlow B. Bizon J. L. (2018). Age-related declines in prefrontal cortical expression of metabotropic glutamate receptors that support working memory. eNeuro 5. doi: 10.1523/ENEURO.0164-18.2018, 29971246 PMC6026020

[ref101] Hill R. S. Walsh C. A. (2005). Molecular insights into human brain evolution. Nature 437, 64–67. doi: 10.1038/nature04103, 16136130

[ref102] Hof P. R. Morrison J. H. (1991). Neocortical neuronal subpopulations labeled by a monoclonal antibody to calbindin exhibit differential vulnerability in Alzheimer's disease. Exp. Neurol. 111, 293–301. doi: 10.1016/0014-4886(91)90096-u, 1999232

[ref103] Holtzman D. M. Herz J. Bu G. (2012). Apolipoprotein E and apolipoprotein E receptors: normal biology and roles in Alzheimer disease. Cold Spring Harb. Perspect. Med. 2:a006312. doi: 10.1101/cshperspect.a006312, 22393530 PMC3282491

[ref104] Hyman B. T. Van Hoesen G. W. Damasio A. R. Barnes C. L. (1984). Alzheimer's disease: cell-specific pathology isolates the hippocampal formation. Science 225, 1168–1170. doi: 10.1126/science.6474172, 6474172

[ref105] Iguchi A. Takatori S. Kimura S. Muneto H. Wang K. Etani H. . (2023). INPP5D modulates TREM2 loss-of-function phenotypes in a β-amyloidosis mouse model. iScience 26:106375. doi: 10.1016/j.isci.2023.106375, 37035000 PMC10074152

[ref106] Jin L. E. Wang M. Galvin V. C. Lightbourne T. C. Conn P. J. Arnsten A. F. T. . (2018). mGluR2 versus mGluR3 metabotropic glutamate receptors in primate dorsolateral prefrontal cortex: postsynaptic mGluR3 strengthen working memory networks. Cereb. Cortex 28, 974–987. doi: 10.1093/cercor/bhx005, 28108498 PMC5974790

[ref107] Jin L. E. Wang M. Yang S. T. Yang Y. Galvin V. C. Lightbourne T. C. . (2017). mGluR2/3 mechanisms in primate dorsolateral prefrontal cortex: evidence for both presynaptic and postsynaptic actions. Mol. Psychiatry 22, 1615–1625. doi: 10.1038/mp.2016.129, 27502475 PMC5298940

[ref108] Jorfi M. Park J. Hall C. K. Lin C. J. Chen M. von Maydell D. . (2023). Infiltrating CD8(+) T cells exacerbate Alzheimer's disease pathology in a 3D human neuroimmune axis model. Nat. Neurosci. 26, 1489–1504. doi: 10.1038/s41593-023-01415-3, 37620442 PMC11184920

[ref109] Joyce M. K. P. Garcia-Cabezas M. A. John Y. J. Barbas H. (2020). Serial prefrontal pathways are positioned to balance cognition and emotion in Primates. J. Neurosci. 40, 8306–8328. doi: 10.1523/JNEUROSCI.0860-20.2020, 32989097 PMC7577604

[ref110] Kaufman S. K. Del Tredici K. Thomas T. L. Braak H. Diamond M. I. (2018). Tau seeding activity begins in the transentorhinal/entorhinal regions and anticipates phospho-tau pathology in Alzheimer's disease and PART. Acta Neuropathol. 136, 57–67. doi: 10.1007/s00401-018-1855-6, 29752551 PMC6015098

[ref111] Kelliher M. Fastbom J. Cowburn R. F. Bonkale W. Ohm T. G. Ravid R. . (1999). Alterations in the ryanodine receptor calcium release channel correlate with Alzheimer's disease neurofibrillary and beta-amyloid pathologies. Neuroscience 92, 499–513. doi: 10.1016/s0306-4522(99)00042-1, 10408600

[ref112] Khachaturian Z. S. (1991). Overview of basic research on Alzheimer disease: implications for cognition. Alzheimer Dis. Assoc. Disord. 5, S1–S6. doi: 10.1097/00002093-199100051-00002 1664213

[ref113] Kindler J. Lim C. K. Weickert C. S. Boerrigter D. Galletly C. Liu D. . (2020). Dysregulation of kynurenine metabolism is related to proinflammatory cytokines, attention, and prefrontal cortex volume in schizophrenia. Mol. Psychiatry 25, 2860–2872. doi: 10.1038/s41380-019-0401-9, 30940904 PMC7577855

[ref114] King A. (2018). The search for better animal models of Alzheimer's disease. Nature 559, S13–S15. doi: 10.1038/d41586-018-05722-9, 30046083

[ref115] Kodamullil A. T. Iyappan A. Karki R. Madan S. Younesi E. Hofmann-Apitius M. (2017). Of mice and men: comparative analysis of neuro-inflammatory mechanisms in human and mouse using cause-and-effect models. J Alzheimer's Dis 59, 1045–1055. doi: 10.3233/jad-170255, 28731442 PMC5545904

[ref116] Kondo H. Tanaka K. Hashikawa T. Jones E. G. (1999). Neurochemical gradients along monkey sensory cortical pathways: calbindin-immunoreactive pyramidal neurons in layers II and III. Eur. J. Neurosci. 11, 4197–4203, 10594645 10.1046/j.1460-9568.1999.00844.x

[ref117] Kucukkilic E. Brookes K. Barber I. Guetta-Baranes T. A. Consortium Morgan K. . (2018). Complement receptor 1 gene (CR1) intragenic duplication and risk of Alzheimer's disease. Hum. Genet. 137, 305–314. doi: 10.1007/s00439-018-1883-2 29675612 PMC5937907

[ref118] Lacampagne A. Liu X. Reiken S. Bussiere R. Meli A. C. Lauritzen I. . (2017). Post-translational remodeling of ryanodine receptor induces calcium leak leading to Alzheimer's disease-like pathologies and cognitive deficits. Acta Neuropathol. 134, 749–767. doi: 10.1007/s00401-017-1733-7, 28631094

[ref119] LaFerla F. M. Green K. N. Oddo S. (2007). Intracellular amyloid-beta in Alzheimer's disease. Nat. Rev. Neurosci. 8, 499–509. doi: 10.1038/nrn2168 17551515

[ref120] Larramona-Arcas R. Gonzalez-Arias C. Perea G. Gutierrez A. Vitorica J. Garcia-Barrera T. . (2020). Sex-dependent calcium hyperactivity due to lysosomal-related dysfunction in astrocytes from APOE4 versus APOE3 gene targeted replacement mice. Mol. Neurodegener. 15:35. doi: 10.1186/s13024-020-00382-8, 32517777 PMC7285605

[ref121] Lewis D. A. Campbell M. J. Terry R. D. Morrison J. H. (1987). Laminar and regional distributions of neurofibrillary tangles and neuritic plaques in Alzheimer's disease: a quantitative study of visual and auditory cortices. J. Neurosci. 7, 1799–1808. doi: 10.1523/jneurosci.07-06-01799.1987, 2439665 PMC6568896

[ref122] Leyane T. S. Jere S. W. Houreld N. N. (2022). Oxidative stress in ageing and chronic degenerative pathologies: molecular mechanisms involved in counteracting oxidative stress and chronic inflammation. Int. J. Mol. Sci. 23. doi: 10.3390/ijms23137273, 35806275 PMC9266760

[ref123] Li J. T. Xie X. M. Yu J. Y. Sun Y. X. Liao X. M. Wang X. X. . (2017). Suppressed Calbindin levels in hippocampal excitatory neurons mediate stress-induced memory loss. Cell Rep. 21, 891–900. doi: 10.1016/j.celrep.2017.10.006, 29069596

[ref124] Liddelow S. A. Guttenplan K. A. Clarke L. E. Bennett F. C. Bohlen C. J. Schirmer L. . (2017). Neurotoxic reactive astrocytes are induced by activated microglia. Nature 541, 481–487. doi: 10.1038/nature21029, 28099414 PMC5404890

[ref125] Lish A. M. Grogan E. F. L. Benoit C. R. Pearse R. V. 2nd Heuer S. E. Luquez T. . (2025). CLU alleviates Alzheimer's disease-relevant processes by modulating astrocyte reactivity and microglia-dependent synaptic density. Neuron 113, 1925–1946 e11. doi: 10.1016/j.neuron.2025.03.034, 40311610 PMC12181066

[ref126] Liu C. C. Zhao N. Fu Y. Wang N. Linares C. Tsai C. W. . (2017). ApoE4 accelerates early seeding of amyloid pathology. Neuron 96, 1024–1032.e3. doi: 10.1016/j.neuron.2017.11.013, 29216449 PMC5948105

[ref127] MacKenzie K. F. Wallace D. A. Hill E. V. Anthony D. F. Henderson D. J. Houslay D. M. . (2011). Phosphorylation of cAMP-specific PDE4A5 (phosphodiesterase-4A5) by MK2 (MAPKAPK2) attenuates its activation through protein kinase a phosphorylation. Biochem. J. 435, 755–769. doi: 10.1042/BJ20101184, 21323643

[ref128] Magrou L. Joyce M. K. P. Froudist-Walsh S. Datta D. Wang X. J. Martinez-Trujillo J. . (2024). The meso-connectomes of mouse, marmoset, and macaque: network organization and the emergence of higher cognition. Cereb. Cortex 34:bhae174. doi: 10.1093/cercor/bhae174, 38771244 PMC11107384

[ref129] Malik M. Simpson J. F. Parikh I. Wilfred B. R. Fardo D. W. Nelson P. T. . (2013). CD33 Alzheimer's risk-altering polymorphism, CD33 expression, and exon 2 splicing. J. Neurosci. 33, 13320–13325. doi: 10.1523/JNEUROSCI.1224-13.2013, 23946390 PMC3742922

[ref130] Mandelkow E. M. Mandelkow E. (1998). Tau in Alzheimer's disease. Trends Cell Biol. 8, 425–427. doi: 10.1016/s0962-8924(98)01368-3, 9854307

[ref131] Martens Y. A. Zhao N. Liu C. C. Kanekiyo T. Yang A. J. Goate A. M. . (2022). ApoE Cascade hypothesis in the pathogenesis of Alzheimer's disease and related dementias. Neuron 110, 1304–1317. doi: 10.1016/j.neuron.2022.03.004, 35298921 PMC9035117

[ref132] Marx S. O. Reiken S. Hisamatsu Y. Jayaraman T. Burkhoff D. Rosemblit N. . (2000). PKA phosphorylation dissociates FKBP12.6 from the calcium release channel (ryanodine receptor): defective regulation in failing hearts. Cell 101, 365–376, 10830164 10.1016/s0092-8674(00)80847-8

[ref133] Matsuo E. S. Shin R. W. Billingsley M. L. Van deVoorde A. O'Connor M. Trojanowski J. Q. . (1994). Biopsy-derived adult human brain tau is phosphorylated at many of the same sites as Alzheimer's disease paired helical filament tau. Neuron 13, 989–1002. doi: 10.1016/0896-6273(94)90264-x, 7946342

[ref134] Mattson M. P. (2007). Calcium and neurodegeneration. Aging Cell 6, 337–350. doi: 10.1111/j.1474-9726.2007.00275.x, 17328689

[ref135] Mendes A. J. Ribaldi F. Lathuiliere A. Ashton N. J. Janelidze S. Zetterberg H. . (2024). Head-to-head study of diagnostic accuracy of plasma and cerebrospinal fluid p-tau217 versus p-tau181 and p-tau231 in a memory clinic cohort. J. Neurol. 271, 2053–2066. doi: 10.1007/s00415-023-12148-5, 38195896 PMC10972950

[ref136] Mishra S. Blazey T. M. Holtzman D. M. Cruchaga C. Su Y. Morris J. C. . (2018). Longitudinal brain imaging in preclinical Alzheimer disease: impact of APOE ε4 genotype. Brain 141, 1828–1839. doi: 10.1093/brain/awy103, 29672664 PMC5972633

[ref137] Monosov I. Haber S. N. Leuthardt E. C. Jezzini A. (2020). Anterior cingulate cortex and the control of dynamic behavior in primates. Current Bio 30, R1442–R1454. doi: 10.1016/j.cub.2020.10.009, 33290716 PMC8197026

[ref138] Morrison C. Dadar M. Kamal F. Collins D. L. (2024). Differences in Alzheimer's disease-related pathology profiles across apolipoprotein groups. J. Gerontol. A Biol. Sci. Med. Sci. 79. doi: 10.1093/gerona/glad254, 37935216 PMC10799756

[ref139] Mufson E. J. Benzing W. C. Cole G. M. Wang H. Emerich D. F. Sladek J. R. J. . (1994). Apolipoprotein E-immunoreactivity in aged rhesus monkey cortex: colocalization with amyloid plaques. Neurobiol. Aging 15, 621–627. doi: 10.1016/0197-4580(94)00064-6, 7824054

[ref140] Muntané G. Horvath J. E. Hof P. R. Ely J. J. Hopkins W. D. Raghanti M. A. . (2015). Analysis of synaptic gene expression in the neocortex of primates reveals evolutionary changes in glutamatergic neurotransmission. Cereb. Cortex 25, 1596–1607. doi: 10.1093/cercor/bht354, 24408959 PMC4428301

[ref141] Murphy K. R. Landau S. M. Choudhury K. R. Hostage C. A. Shpanskaya K. S. Sair H. I. . (2013). Disease neuroimaging: mapping the effects of ApoE4, age and cognitive status on 18F-florbetapir PET measured regional cortical patterns of beta-amyloid density and growth. NeuroImage 78, 474–480. doi: 10.1016/j.neuroimage.2013.04.048, 23624169 PMC3749874

[ref142] Murray J. D. Bernacchia A. Freedman D. J. Romo R. Wallis J. D. Cai X. . (2014). A hierarchy of intrinsic timescales across primate cortex. Nat. Neurosci. 17, 1661–1663. doi: 10.1038/nn.3862, 25383900 PMC4241138

[ref143] Nelson P. T. Alafuzoff I. Bigio E. H. Bouras C. Braak H. Cairns N. J. . (2012). Correlation of Alzheimer disease neuropathologic changes with cognitive status: a review of the literature. J. Neuropathol. Exp. Neurol. 71, 362–381. doi: 10.1097/NEN.0b013e31825018f7, 22487856 PMC3560290

[ref144] Nikolaienko R. Bovo E. Zima A. V. (2018). Redox dependent modifications of ryanodine receptor: basic mechanisms and implications in heart diseases. Front. Physiol. 9:1775. doi: 10.3389/fphys.2018.01775, 30574097 PMC6291498

[ref145] Norgaard C. H. Friedrich S. Hansen C. T. Gerds T. Ballard C. Moller D. V. . (2022). Treatment with glucagon-like peptide-1 receptor agonists and incidence of dementia: data from pooled double-blind randomized controlled trials and nationwide disease and prescription registers. Alzheimers Dement (N Y) 8:e12268. doi: 10.1002/trc2.12268, 35229024 PMC8864443

[ref146] Nowak L. Bregestovski P. Ascher P. Herbet A. Prochiantz A. (1984). Magnesium gates glutamate-activated channels in mouse central neurones. Nature 307, 462–465. doi: 10.1038/307462a0, 6320006

[ref147] Nugent A. A. Lin K. van Lengerich B. Lianoglou S. Przybyla L. Davis S. S. . (2020). TREM2 regulates microglial cholesterol metabolism upon chronic phagocytic challenge. Neuron 105, 837–854.e9. doi: 10.1016/j.neuron.2019.12.007, 31902528

[ref148] Ohkubo N. Mitsuda N. Tamatani M. Yamaguchi A. Lee Y. D. Ogihara T. . (2001). Apolipoprotein E4 stimulates cAMP response element-binding protein transcriptional activity through the extracellular signal-regulated kinase pathway. J. Biol. Chem. 276, 3046–3053. doi: 10.1074/jbc.M005070200 11042199

[ref149] Ohm T. G. Hamker U. Cedazo-Minguez A. Röckl W. Scharnagl H. März W. . (2001). Apolipoprotein E and beta A4-amyloid: signals and effects. Biochem. Soc. Symp. 67, 121–129. doi: 10.1042/bss0670121 11447828

[ref150] Olszewski R. T. Janczura K. J. Bzdega T. Der E. K. Venzor F. O'Rourke B. . (2017). NAAG peptidase inhibitors act via mGluR3: animal models of memory, Alzheimer's, and ethanol intoxication. Neurochem. Res. 42, 2646–2657. doi: 10.1007/s11064-017-2181-4, 28285415 PMC5603630

[ref151] Orhan F. Malwade S. Khanlarkhani N. Gkogka A. Langeder A. Jungholm O. . (2025). Kynurenic acid and promotion of activity-dependent synapse elimination in schizophrenia. Am. J. Psychiatry 182, 389–400. doi: 10.1176/appi.ajp.20240048, 40165559

[ref152] Palmqvist S. Janelidze S. Quiroz Y. T. Zetterberg H. Lopera F. Stomrud E. . (2020). Discriminative accuracy of plasma Phospho-tau217 for Alzheimer disease vs other neurodegenerative disorders. JAMA 324, 772–781. doi: 10.1001/jama.2020.12134, 32722745 PMC7388060

[ref153] Pandey N. Yang Z. Cieza B. Reyes-Dumeyer D. Kang M. S. Montesinos R. . (2025). Plasma phospho-tau217 as a predictive biomarker for Alzheimer's disease in a large south American cohort. Alzheimer's Res Ther 17:1. doi: 10.1186/s13195-024-01655-w, 39743558 PMC11694372

[ref154] Parhizkar S. Arzberger T. Brendel M. Kleinberger G. Deussing M. Focke C. . (2019). Loss of TREM2 function increases amyloid seeding but reduces plaque-associated ApoE. Nat. Neurosci. 22, 191–204. doi: 10.1038/s41593-018-0296-9, 30617257 PMC6417433

[ref155] Paspalas C. D. Carlyle B. C. Leslie S. Preuss T. M. Crimins J. L. Huttner A. J. . (2018). The aged rhesus macaque manifests Braak stage III/IV Alzheimer's-like pathology. Alzheimers Dement. 14, 680–691. doi: 10.1016/j.jalz.2017.11.005, 29241829 PMC6178089

[ref156] Perlmutter L. S. Scott S. A. Barron E. Chui H. C. (1992). MHC class II-positive microglia in human brain: association with Alzheimer lesions. J. Neurosci. Res. 33, 549–558. doi: 10.1002/jnr.490330407, 1484388

[ref157] Pires M. Rego A. C. (2023). Apoe4 and Alzheimer's disease pathogenesis-mitochondrial deregulation and targeted therapeutic strategies. Int. J. Mol. Sci. 24. doi: 10.3390/ijms24010778, 36614219 PMC9821307

[ref158] Preuss T. M. (2012). Human brain evolution: from gene discovery to phenotype discovery. Proc. Natl. Acad. Sci. USA 109 Suppl 1, 10709–10716. doi: 10.1073/pnas.1201894109, 22723367 PMC3386880

[ref159] Ramakrishna S. Jhaveri V. Konings S. C. Nawalpuri B. Chakraborty S. Holst B. . (2021). APOE4 affects basal and NMDAR-mediated protein synthesis in neurons by perturbing calcium homeostasis. J. Neurosci. 41, 8686–8709. doi: 10.1523/JNEUROSCI.0435-21.2021, 34475200 PMC8528497

[ref160] Ramos B. P. Birnbaum S. G. Lindenmayer I. Newton S. S. Duman R. S. Arnsten A. F. (2003). Dysregulation of protein kinase a signaling in the aged prefrontal cortex: new strategy for treating age-related cognitive decline. Neuron 40, 835–845. doi: 10.1016/s0896-6273(03)00694-9, 14622586

[ref161] Rapp P. R. Amaral D. G. (1989). Evidence for task-dependent memory dysfunction in the aged monkey. J. Neurosci. 9, 3568–3576. doi: 10.1523/jneurosci.09-10-03568.1989, 2795141 PMC6569903

[ref162] Rauch J. N. Luna G. Guzman E. Audouard M. Challis C. Sibih Y. E. . (2020). LRP1 is a master regulator of tau uptake and spread. Nature 580, 381–385. doi: 10.1038/s41586-020-2156-5, 32296178 PMC7687380

[ref163] Raulin A. C. Doss S. V. Trottier Z. A. Ikezu T. C. Bu G. Liu C. C. (2022). ApoE in Alzheimer's disease: pathophysiology and therapeutic strategies. Mol. Neurodegener. 17:72. doi: 10.1186/s13024-022-00574-4, 36348357 PMC9644639

[ref164] Reading C. L. Ahlem C. N. Murphy M. F. (2021). NM101 phase III study of NE3107 in Alzheimer's disease: rationale, design and therapeutic modulation of neuroinflammation and insulin resistance. Neurodegener Dis Manag 11, 289–298. doi: 10.2217/nmt-2021-0022, 34251287

[ref165] Reiken S. Sittenfeld L. Dridi H. Liu Y. Liu X. Marks A. R. (2022). Alzheimer's-like signaling in brains of COVID-19 patients. Alzheimers Dement. 18, 955–965. doi: 10.1002/alz.12558, 35112786 PMC9011576

[ref166] Rissman R. A. Poon W. W. Blurton-Jones M. Oddo S. Torp R. Vitek M. P. . (2004). Caspase-cleavage of tau is an early event in Alzheimer disease tangle pathology. J. Clin. Invest. 114, 121–130. doi: 10.1172/JCI20640, 15232619 PMC437967

[ref167] Rodriguez-Callejas J. D. Fuchs E. Perez-Cruz C. (2016). Evidence of tau hyperphosphorylation and dystrophic microglia in the common marmoset. Front. Aging Neurosci. 8:315. doi: 10.3389/fnagi.2016.00315, 28066237 PMC5177639

[ref168] Rosenzweig N. Kleemann K. L. Rust T. Carpenter M. Grucci M. Aronchik M. . (2024). Sex-dependent APOE4 neutrophil-microglia interactions drive cognitive impairment in Alzheimer's disease. Nat. Med. 30, 2990–3003. doi: 10.1038/s41591-024-03122-3, 38961225 PMC12952287

[ref169] Sabogal-Guaqueta A. M. Marmolejo-Garza A. Trombetta-Lima M. Oun A. Hunneman J. Chen T. . (2023). Species-specific metabolic reprogramming in human and mouse microglia during inflammatory pathway induction. Nat. Commun. 14:6454. doi: 10.1038/s41467-023-42096-7, 37833292 PMC10575978

[ref170] Samuels J. D. Moore K. A. Ennerfelt H. E. Johnson A. M. Walsh A. E. Price R. J. . (2023). The Alzheimer's disease risk factor INPP5D restricts neuroprotective microglial responses in amyloid beta-mediated pathology. Alzheimers Dement. 19, 4908–4921. doi: 10.1002/alz.13089, 37061460 PMC10576836

[ref171] Schwarcz R. Rassoulpour A. Wu H. Q. Medoff D. Tamminga C. A. Roberts R. C. (2001). Increased cortical kynurenate content in schizophrenia. Biol. Psychiatry 50, 521–530. doi: 10.1016/s0006-3223(01)01078-2, 11600105

[ref172] Serrano-Pozo A. Qian J. Monsell S. E. Betensky R. A. Hyman B. T. (2015). APOEε2 is associated with milder clinical and pathological Alzheimer disease. Ann. Neurol. 77, 917–929. doi: 10.1002/ana.24369, 25623662 PMC4447539

[ref173] Sharma G. Huo A. Kimura T. Shiozawa S. Kobayashi R. Sahara N. . (2019). Tau isoform expression and phosphorylation in marmoset brains. J. Biol. Chem. 294, 11433–11444. doi: 10.1074/jbc.RA119.008415, 31171723 PMC6663862

[ref174] Sheffield L. G. Berman N. E. (1998). Microglial expression of MHC class II increases in normal aging of nonhuman primates. Neurobiol. Aging 19, 47–55. doi: 10.1016/s0197-4580(97)00168-1, 9562503

[ref175] Shi Y. Manis M. Long J. Wang K. Sullivan P. M. Remolina Serrano J. . (2019). Microglia drive APOE-dependent neurodegeneration in a tauopathy mouse model. J. Exp. Med. 216, 2546–2561. doi: 10.1084/jem.20190980, 31601677 PMC6829593

[ref176] Sousa A. M. M. Meyer K. A. Santpere G. Gulden F. O. Sestan N. (2017). Evolution of the human nervous system function, structure, and development. Cell 170, 226–247. doi: 10.1016/j.cell.2017.06.036, 28708995 PMC5647789

[ref177] Spatharas P. M. Nasi G. I. Tsiolaki P. L. Theodoropoulou M. K. Papandreou N. C. Hoenger A. . (2022). Clusterin in Alzheimer's disease: An amyloidogenic inhibitor of amyloid formation? Biochim. Biophys. Acta Mol. basis Dis. 1868:166384. doi: 10.1016/j.bbadis.2022.166384, 35292360

[ref178] Stephan A. H. Barres B. A. Stevens B. (2012). The complement system: an unexpected role in synaptic pruning during development and disease. Annu. Rev. Neurosci. 35, 369–389. doi: 10.1146/annurev-neuro-061010-113810, 22715882

[ref179] Stephan A. H. Madison D. V. Mateos J. M. Fraser D. A. Lovelett E. A. Coutellier L. . (2013). A dramatic increase of C1q protein in the CNS during normal aging. J. Neurosci. 33, 13460–13474. doi: 10.1523/JNEUROSCI.1333-13.2013, 23946404 PMC3742932

[ref180] Stone T. W. Addae J. I. (2002). The pharmacological manipulation of glutamate receptors and neuroprotection. Eur. J. Pharmacol. 447, 285–296. doi: 10.1016/s0014-2999(02)01851-4, 12151020

[ref181] Stone T. W. Williams R. O. (2023). Modulation of T cells by tryptophan metabolites in the kynurenine pathway. Trends Pharmacol. Sci. 44, 442–456. doi: 10.1016/j.tips.2023.04.006, 37248103

[ref182] Strittmatter W. J. Saunders A. M. Schmechel D. Pericak-Vance M. Enghild J. Salvesen G. S. . (1993). Apolipoprotein E: high-avidity binding to beta-amyloid and increased frequency of type 4 allele in late-onset familial Alzheimer disease. Proc. Natl. Acad. Sci. USA 90, 1977–1981. doi: 10.1073/pnas.90.5.1977, 8446617 PMC46003

[ref183] Stutzmann G. E. (2005). Calcium dysregulation, IP3 signaling, and Alzheimer's disease. Neuroscientist 11, 110–115. doi: 10.1177/1073858404270899, 15746379

[ref184] Stutzmann G. E. (2007). The pathogenesis of Alzheimers disease is it a lifelong "calciumopathy"? Neuroscientist 13, 546–559. doi: 10.1177/1073858407299730, 17901262

[ref185] Stutzmann G. E. Caccamo A. LaFerla F. M. Parker I. (2004). Dysregulated IP3 signaling in cortical neurons of knock-in mice expressing an Alzheimer's-linked mutation in presenilin1 results in exaggerated Ca2+ signals and altered membrane excitability. J. Neurosci. 24, 508–513. doi: 10.1523/JNEUROSCI.4386-03.2004, 14724250 PMC6729995

[ref186] Swardfager W. Lanctot K. Rothenburg L. Wong A. Cappell J. Herrmann N. (2010). A meta-analysis of cytokines in Alzheimer's disease. Biol. Psychiatry 68, 930–941. doi: 10.1016/j.biopsych.2010.06.012, 20692646

[ref187] Thibault O. Hadley R. Landfield P. W. (2001). Elevated postsynaptic [Ca2+]i and L-type calcium channel activity in aged hippocampal neurons: relationship to impaired synaptic plasticity. J. Neurosci. 21, 9744–9756. doi: 10.1523/JNEUROSCI.21-24-09744.2001, 11739583 PMC6763040

[ref188] Thibault O. Landfield P. W. (1996). Increase in single L-type calcium channels in hippocampal neurons during aging. Science 272, 1017–1020. doi: 10.1126/science.272.5264.1017, 8638124

[ref189] Tsai A. P. Lin P. B. Dong C. Moutinho M. Casali B. T. Liu Y. . (2021). INPP5D expression is associated with risk for Alzheimer's disease and induced by plaque-associated microglia. Neurobiol. Dis. 153:105303. doi: 10.1016/j.nbd.2021.105303, 33631273 PMC8082515

[ref190] Ueda K. Shinohara S. Yagami T. Asakura K. Kawasaki K. (1997). Amyloid beta protein potentiates Ca2+ influx through L-type voltage-sensitive Ca2+ channels: a possible involvement of free radicals. J. Neurochem. 68, 265–271. doi: 10.1046/j.1471-4159.1997.68010265.x, 8978734

[ref191] Ulland T. K. Colonna M. (2018). TREM2 - a key player in microglial biology and Alzheimer disease. Nat. Rev. Neurol. 14, 667–675. doi: 10.1038/s41582-018-0072-1, 30266932

[ref192] Um J. W. Kaufman A. C. Kostylev M. Heiss J. K. Stagi M. Takahashi H. . (2013). Metabotropic glutamate receptor 5 is a coreceptor for Alzheimer aβ oligomer bound to cellular prion protein. Neuron 79, 887–902. doi: 10.1016/j.neuron.2013.06.036, 24012003 PMC3768018

[ref193] Uno H. Walker L. C. (1993). The age of biosenescence and the incidence of cerebral beta-amyloidosis in aged captive rhesus monkeys. Ann. N. Y. Acad. Sci. 695, 232–235. doi: 10.1111/j.1749-6632.1993.tb23058.x, 8239288

[ref194] Valiukas Z. Tangalakis K. Apostolopoulos V. Feehan J. (2025). Microglial activation states and their implications for Alzheimer's disease. J. Prev Alzheimers Dis. 12:100013. doi: 10.1016/j.tjpad.2024.100013, 39800461 PMC12184064

[ref195] van Dyck C. H. Swanson C. J. Aisen P. Bateman R. J. Chen C. Gee M. . (2023). Lecanemab in early Alzheimer's disease. N. Engl. J. Med. 388, 9–21. doi: 10.1056/NEJMoa2212948, 36449413

[ref196] Vanderlind W. M. Rabinovitz B. B. Miao I. Y. Oberlin L. E. Bueno-Castellano C. Fridman C. . (2021). A systematic review of neuropsychological and psychiatric sequalae of COVID-19: implications for treatment. Curr. Opin. Psychiatry 34, 420–433. doi: 10.1097/YCO.0000000000000713, 34016818 PMC8183238

[ref197] Vijayraghavan S. Wang M. Birnbaum S. G. Williams G. V. Arnsten A. F. (2007). Inverted-U dopamine D1 receptor actions on prefrontal neurons engaged in working memory. Nat. Neurosci. 10, 376–384. doi: 10.1038/nn1846, 17277774

[ref198] Wang S. Li B. Solomon V. Fonteh A. Rapoport S. I. Bennett D. A. . (2022). Calcium-dependent cytosolic phospholipase a(2) activation is implicated in neuroinflammation and oxidative stress associated with ApoE4. Mol. Neurodegener. 17:42. doi: 10.1186/s13024-022-00549-5, 35705959 PMC9202185

[ref199] Wang S. Mustafa M. Yuede C. M. Salazar S. V. Kong P. Long H. . (2020). Anti-human TREM2 induces microglia proliferation and reduces pathology in an Alzheimer's disease model. J. Exp. Med. 217. doi: 10.1084/jem.20200785, 32579671 PMC7478730

[ref200] Wang S. Sudan R. Peng V. Zhou Y. Du S. Yuede C. M. . (2022). TREM2 drives microglia response to amyloid-beta via SYK-dependent and -independent pathways. Cell 185, 4153–4169.e19. doi: 10.1016/j.cell.2022.09.033, 36306735 PMC9625082

[ref201] Wang M. Yang Y. Wang C. J. Gamo N. J. Jin L. E. Mazer J. A. . (2013). NMDA receptors subserve persistent neuronal firing during working memory in dorsolateral prefrontal cortex. Neuron 77, 736–749. doi: 10.1016/j.neuron.2012.12.032, 23439125 PMC3584418

[ref202] Wang Y. Zhang Y. Hu W. Xie S. Gong C. X. Iqbal K. . (2015). Rapid alteration of protein phosphorylation during postmortem: implication in the study of protein phosphorylation. Sci. Rep. 5:15709. doi: 10.1038/srep15709, 26511732 PMC4625177

[ref203] Widner B. Leblhuber F. Walli J. Tilz G. P. Demel U. Fuchs D. (2000). Tryptophan degradation and immune activation in Alzheimer's disease. J. Neural Transm. 107, 343–353. doi: 10.1007/s007020050029, 10821443

[ref204] Willis M. Kaufmann W. A. Wietzorrek G. Hutter-Paier B. Moosmang S. Humpel C. . (2010). L-type calcium channel CaV 1.2 in transgenic mice overexpressing human AbetaPP751 with the London (V717I) and Swedish (K670M/N671L) mutations. J Alzheimer's Dis 20, 1167–1180. doi: 10.3233/JAD-2010-091117, 20413896

[ref205] Woo E. Datta D. Arnsten A. F. T. (2022). Glutamate metabotropic receptor type 3 (mGlu3) localization in the rat Prelimbic medial prefrontal cortex. Front. Neuroanat. 16:849937. doi: 10.3389/fnana.2022.849937, 35444520 PMC9013768

[ref206] Woo E. Sansing L. H. Arnsten A. F. T. Datta D. (2021). Chronic stress weakens connectivity in the prefrontal cortex: architectural and molecular changes. Chronic Stress (Thousand Oaks) 5:24705470211029254. doi: 10.1177/24705470211029254, 34485797 PMC8408896

[ref207] Yang S. Datta D. Elizabeth W. Duque A. Morozov Y. M. Arellano J. . (2022). Inhibition of glutamate-carboxypeptidase-II in dorsolateral prefrontal cortex: potential therapeutic target for neuroinflammatory cognitive disorders. Mol. Psychiatry 27, 4252–4263. doi: 10.1038/s41380-022-01656-x, 35732693 PMC9718677

[ref208] Yang S. Datta D. Krienen F. M. Ling E. Woo E. May A. . (2024). Kynurenic acid inflammatory signaling expands in primates and impairs prefrontal cortical cognition. bioRxiv. doi: 10.1101/2024.06.13.598842, 41413200 PMC12929064

[ref209] Yang Y. Paspalas C. D. Jin L. E. Picciotto M. R. Arnsten A. F. Wang M. (2013). Nicotinic alpha7 receptors enhance NMDA cognitive circuits in dorsolateral prefrontal cortex. Proc. Natl. Acad. Sci. USA 110, 12078–12083. doi: 10.1073/pnas.1307849110, 23818597 PMC3718126

[ref210] Yin Z. Rosenzweig N. Kleemann K. L. Zhang X. Brandão W. Margeta M. A. . (2023). APOE4 impairs the microglial response in Alzheimer's disease by inducing TGFβ-mediated checkpoints. Nat. Immunol. 24, 1839–1853. doi: 10.1038/s41590-023-01627-6, 37749326 PMC10863749

[ref211] Young M. E. Ohm D. T. Dumitriu D. Rapp P. R. Morrison J. H. (2014). Differential effects of aging on dendritic spines in visual cortex and prefrontal cortex of the rhesus monkey. Neuroscience 274, 33–43. doi: 10.1016/j.neuroscience.2014.05.008, 24853052 PMC4108992

[ref212] Yu J. T. Tan L. (2012). The role of clusterin in Alzheimer's disease: pathways, pathogenesis, and therapy. Mol. Neurobiol. 45, 314–326. doi: 10.1007/s12035-012-8237-1, 22274961

[ref213] Zalocusky K. A. Najm R. Taubes A. L. Hao Y. Yoon S. Y. Koutsodendris N. . (2021). Neuronal ApoE upregulates MHC-I expression to drive selective neurodegeneration in Alzheimer's disease. Nat. Neurosci. 24, 786–798. doi: 10.1038/s41593-021-00851-3, 33958804 PMC9145692

[ref214] Zeng J. Liao Z. Yang H. Wang Q. Wu Z. Hua F. . (2024). T cell infiltration mediates neurodegeneration and cognitive decline in Alzheimer's disease. Neurobiol. Dis. 193:106461. doi: 10.1016/j.nbd.2024.106461, 38437992

[ref215] Zhang Z. Bassam B. Thomas A. G. Williams M. Liu J. Nance E. . (2016). Maternal inflammation leads to impaired glutamate homeostasis and up-regulation of glutamate carboxypeptidase II in activated microglia in the fetal/newborn rabbit brain. Neurobiol. Dis. 94, 116–128. doi: 10.1016/j.nbd.2016.06.010, 27326668 PMC5394739

[ref216] Zhang H. Knight C. Chen S. R. W. Bezprozvanny I. (2023). A gating mutation in ryanodine receptor type 2 rescues phenotypes of Alzheimer's disease mouse models by upregulating neuronal autophagy. J. Neurosci. 43, 1441–1454. doi: 10.1523/JNEUROSCI.1820-22.2022, 36627208 PMC9987572

[ref217] Zhang W. Xiao D. Mao Q. Xia H. (2023). Role of neuroinflammation in neurodegeneration development. Signal Transduct. Target. Ther. 8:267. doi: 10.1038/s41392-023-01486-5, 37433768 PMC10336149

[ref218] Zink C. F. Barker P. B. Sawa A. Weinberger D. R. Wang M. Quillian H. . (2020). Association of Missense Mutation in FOLH1 with decreased NAAG levels and impaired working memory circuitry and cognition. Am. J. Psychiatry 177, 1129–1139. doi: 10.1176/appi.ajp.2020.19111152, 33256444

